# Targeting DNGR‐1 with Fangchinoline Elevates Dendritic Cell Antigen Cross‐Presentation‐Mediated Antitumor Immunity in Melanoma

**DOI:** 10.1002/advs.76043

**Published:** 2026-06-11

**Authors:** Yuan Liao, Zhenyang Ren, Jing Zhou, He Luo, Yuling Li, Dan Chen, Yuxiao Jiang, Canzhe Li, Mengyi Zheng, Anqi Li, Yao Wang, Yang Zhang, Dingye Wang, Yanjun Jiang, Jiangyong Gu, Biaoyan Du, Yafei Shi, Fang Liu, Jianyong Xiao, Kun Wang

**Affiliations:** ^1^ Research Center of Integrative Medicine School of Basic Medical Sciences Guangzhou University of Chinese Medicine Guangzhou China; ^2^ Department of Biochemistry Guangzhou University of Chinese Medicine Guangzhou China; ^3^ Research Center of Integrated Chinese and Western Medicine The Affiliated Traditional Chinese Medicine Hospital Southwest Medical University Luzhou China; ^4^ Department of Pathology and Pathophysiology Guangzhou University of Chinese Medicine Guangzhou China; ^5^ Zhongshan Hospital of Traditional Chinese Medicine Affiliated to Guangzhou University of Chinese Medicine Zhongshan China; ^6^ Department of Laboratory Medicine Meishan Tianfu New Area People's Hospital Meishan China; ^7^ Department of Anaesthesia and Intensive Care The Chinese University of Hong Kong Hong Kong SAR China; ^8^ School of Pharmaceutical Sciences Guangzhou University of Chinese Medicine Guangzhou China

**Keywords:** antigen cross‐presentation, cancer immunotherapy, CD8^+^ T cell activation, dendritic cells, DNGR‐1, fangchinoline, Syk–Nox2 signaling

## Abstract

Cross‐presentation of exogenous antigens by dendritic cells (DCs) is essential for initiating CD8^+^ T cell–mediated anti‐tumor immunity. DNGR‐1 (CLEC9A), a C‐type lectin receptor on conventional type 1 DCs (cDC1s), facilitates uptake and cytosolic delivery of dead cell–associated antigens. However, no small‐molecule modulators of DNGR‐1 have been reported. Here, we identify fangchinoline—a natural bisbenzylisoquinoline alkaloid—as a small‐molecule modulator that enhances antigen cross‐presentation in a DNGR‐1‐dependent manner. Functional screening shows fangchinoline promotes MHC‐I–restricted cross‐presentation and CD8^+^ T cell priming. It binds a DNGR‐1 pocket (Trp209, Glu225, Arg226), confirmed by surface plasmon resonance, thermal shift, probe labeling, and mutagenesis. DNGR‐1 engagement activates the Syk–Nox2 pathway, increasing phagosomal reactive oxygen species and membrane permeabilization to enable cytosolic antigen access. Fangchinoline exerts subset‐dependent effects: in GM‐CSF/IL‐4–derived DCs and endogenous cDC1‐associated settings in vivo, it enhances cross‐presentation‐associated function, whereas in Flt3L‐induced DCs with high basal DNGR‐1 expression it promotes DNGR‐1‐dependent cell death. In melanoma models, fangchinoline synergizes with DC transfer and PD‐1 blockade to promote CD8^+^ T cell–mediated tumor control. It enhances antigen‐specific CD8^+^ T cell activation in human DC–T cell co‐cultures. Together, these findings establish fangchinoline as a DNGR‐1‐targeting modulator for context‐dependent enhancement of anti‐tumor immunity.

## Graphical abstract

Fangchinoline is identified as a small‐molecule DNGR‐1 modulator that enhances dendritic‐cell cross‐presentation of tumor antigens. By engaging DNGR‐1 and activating Syk–Nox2 signaling, it promotes phagosomal ROS, antigen escape, MHC‐I presentation, and CD8^+^ T‐cell priming, thereby strengthening antitumor immunity and sensitizing tumors to PD‐1 blockade.

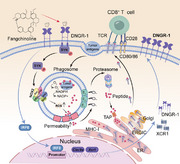

## 1 Introduction

Melanoma, an aggressive malignancy originating from melanocytes, remains one of the most lethal forms of skin cancer worldwide [1]. Although early‐stage disease can often be managed with surgery and chemotherapy, treatment options for advanced or metastatic melanoma are limited, and patient prognosis remains poor [2]. The advent of immune checkpoint inhibitors, particularly monoclonal antibodies targeting programmed cell death protein 1 (PD‐1), its ligand PD‐L1, and cytotoxic T lymphocyte–associated protein 4 (CTLA‐4), has transformed the therapeutic landscape of melanoma [3]. However, only a subset of patients achieves durable responses, and both primary and acquired resistance remain major clinical challenges [4, 5]. These limitations underscore the urgent need for novel immunomodulatory strategies that can enhance the efficacy of existing immunotherapies and overcome tumor‐induced immune suppression [6].

Dendritic cells (DCs) are central regulators of anti‐tumor immunity due to their unique ability to cross‐present exogenous antigens via major histocompatibility complex class I (MHC‐I) molecules, thereby priming cytotoxic CD8^+^ T lymphocytes [7]. This process, known as antigen cross‐presentation, is critical for generating effective T cell–mediated immunity and forms the mechanistic foundation of DC‐based cancer vaccines [8]. Despite its importance, the molecular pathways governing cross‐presentation remain incompletely understood, and few pharmacologic agents have been identified that can specifically enhance this function in DCs [9]. Strategies aimed at modulating DC cross‐presentation represent a promising avenue for amplifying anti‐tumor immune responses both in vaccine development and in combination with checkpoint blockade therapies [10, 11].

C‐type lectin domain family 9 member A (DNGR‐1, also known as CLEC9A) is a dead cell–sensing receptor selectively expressed on conventional type 1 dendritic cells (cDC1s), the DC subset specialized for cross‐priming CD8^+^ T cells [12, 13]. DNGR‐1 recognizes filamentous actin (F‐actin) exposed by necrotic cells and facilitates the uptake and presentation of cell‐associated antigens, playing a non‐redundant role in the initiation of anti‐tumor immunity [12, 14]. This function is tightly regulated by the transcription factor interferon regulatory factor 8 (IRF8), which governs cDC1 lineage specification [12, 15, 16]. Although DNGR‐1 has been extensively studied in genetic models, no small‐molecule modulators capable of directly targeting and activating this receptor have been reported to date, representing a critical gap in our therapeutic armamentarium [17, 18, 19].

Natural products represent a rich and underexplored source of immunomodulatory agents with unique structural and functional properties [20, 21]. In this study, we performed a functional screen of a natural product library using a cell‐based antigen presentation assay and identified fangchinoline—a bisbenzylisoquinoline alkaloid derived from Stephania tetrandra—as a potent enhancer of antigen cross‐presentation in dendritic cells. While fangchinoline has been previously reported to exhibit anti‐proliferative and anti‐inflammatory properties, its immunological mechanisms of action have remained largely unknown [22, 23, 24].

Here, we demonstrate that fangchinoline binds a functional pocket on DNGR‐1—comprising Trp209, Glu225, and Arg226—and enhances downstream signaling through the Syk–Nox2–reactive oxygen species (ROS) axis. This activation promotes phagosomal membrane permeabilization and facilitates the translocation of internalized antigens into the cytosol, a critical step for cross‐presentation via the cytosolic pathway [25, 26]. Notably, fangchinoline selectively enhances cross‐presentation without inducing dendritic cell maturation or inflammatory cytokine release, distinguishing it from conventional immune adjuvants [27]. It further induces selected cross‐presentation‐associated features in GM‐CSF/IL‐4‐derived DCs and synergizes with dendritic cell–based immunotherapy and PD‐1 blockade in murine melanoma models.

By identifying fangchinoline as a small‐molecule functional modulator of DNGR‐1, our study establishes a pharmacological strategy for enhancing dendritic cell antigen‐processing capacity and highlights fangchinoline as a promising candidate for dendritic cell–based immunotherapy. These findings provide a conceptual and mechanistic framework for improving dendritic cell function to elicit robust anti‐tumor responses, with broad translational implications in the context of melanoma and other immunologically responsive malignancies.

## 2 Results

### 2.1 Natural Product‐Based Functional Screening Identifies Fangchinoline as a Potent Enhancer of Antigen Cross‐Presentation and CD8^+^ T Cell Priming

Dendritic cells (DCs) play a central role in the initiation of anti‐tumor immunity by cross‐presenting exogenous antigens to naïve CD8^+^ T cells [28]. However, their functional capacity can be severely impaired in the tumor microenvironment [29]. Exposure of MutuDCs, a murine DC line [30], to B16 melanoma cell–conditioned medium significantly reduced their antigen cross‐presentation ability after 24 h (Figure S1A).

**FIGURE 1:**
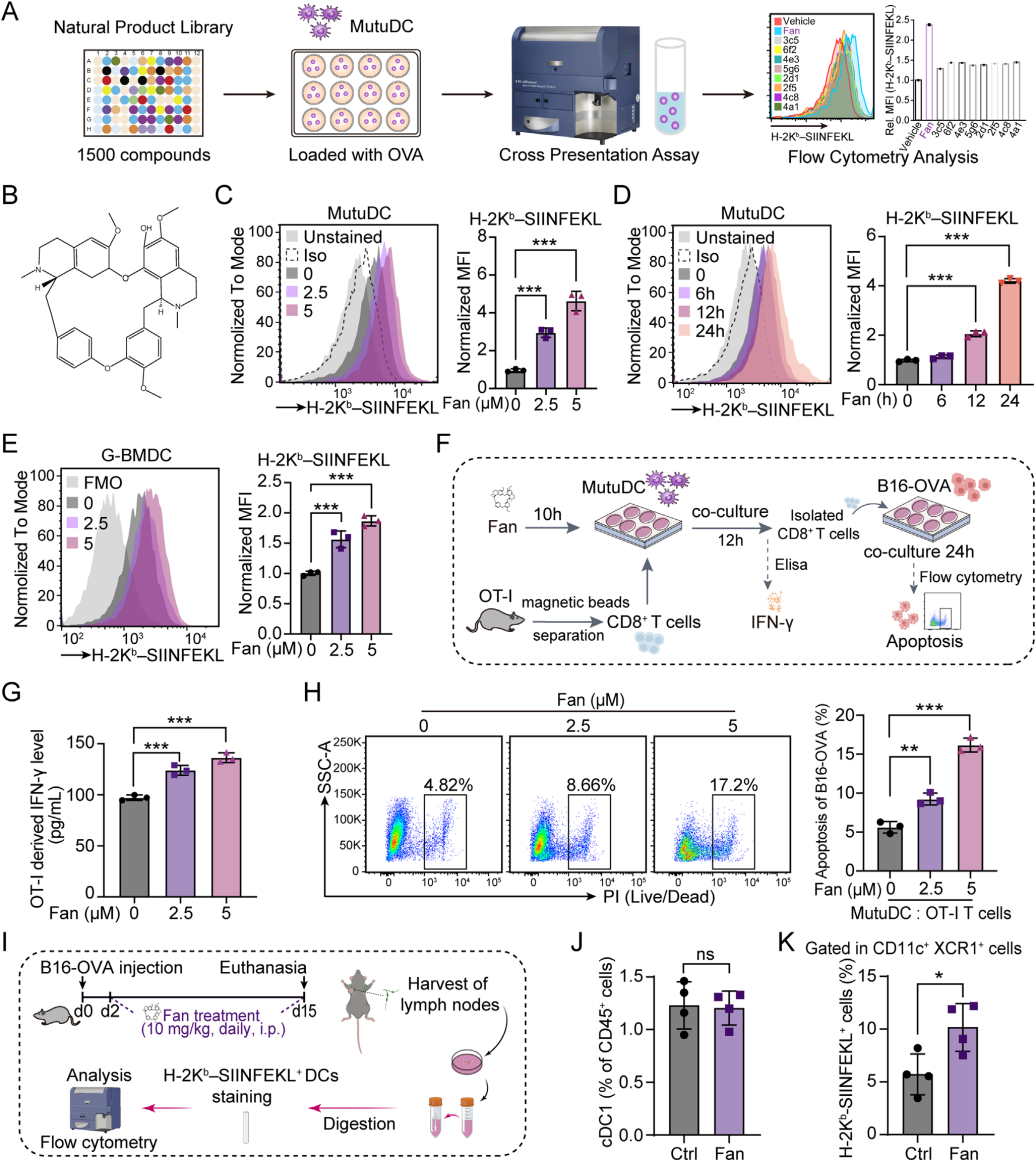
Natural product‐based functional screening identifies fangchinoline as a potent enhancer of antigen cross‐presentation and CD8^+^ T cell priming. (A) To identify small molecules that enhance dendritic cell antigen cross‐presentation, MutuDCs were pulsed with OVA protein (200 µg/mL) and treated with a natural product library for 24 h. Cells were then stained with anti‐mouse H‐2K^b^–SIINFEKL to evaluate mean fluorescence intensity (MFI) via flow cytometry. Fangchinoline was identified as a potent enhancer of antigen cross‐presentation. (B) Chemical structure of fangchinoline. (C) MutuDCs were treated with fangchinoline (2.5 or 5 µM) and OVA protein (200 µg/mL) for 24 h. The MFI of cell‐surface H‐2K^b^–SIINFEKL complexes was measured by flow cytometry. Analyzed by one‐way ANOVA with Tukey's post hoc test (n = 3). (D) Time‐course analysis of antigen cross‐presentation in MutuDCs treated with fangchinoline (5 µM) and OVA (200 µg/mL) for 0, 6, 12, or 24 h. Cross‐presentation was assessed by flow cytometry. Analyzed by one‐way ANOVA with Tukey's post hoc test (n = 3). (E) Bone marrow‐derived dendritic cells (BMDCs) differentiated with GM‐CSF (G‐BMDCs) were treated with fangchinoline (2.5 or 5 µM) and OVA protein (200 µg/mL) for 24 h. Cross‐presentation was measured by flow cytometry. Analyzed by one‐way ANOVA with Tukey's post hoc test (n = 3). (F) Schematic of the in vitro cytotoxicity assay. MutuDCs were pre‐treated with fangchinoline (5 µM) and OVA (200 µg/mL) or vehicle control for 10 h, followed by medium removal and co‐culture with OT‐I CD8^+^ T cells for 12 h. Supernatants were collected for IFN‐γ quantification by ELISA. CD8^+^ T cell‐mediated killing of B16‐OVA target cells was assessed by flow cytometry after 24 h of co‐culture at a 1:5 effector‐to‐target (E:T) ratio. (G) IFN‐γ secretion from OT‐I T cells co‐cultured with fangchinoline‐pretreated MutuDCs (2.5 or 5 µM) was measured by ELISA. Analyzed by one‐way ANOVA with Tukey's post hoc test (n = 3). (H) Cytotoxicity was further evaluated by measuring apoptosis of B16‐OVA cells after co‐culture with OT‐I T cells (primed as in F) at a 1:5 E:T ratio for 24 h. Analyzed by one‐way ANOVA with Tukey's post hoc test (n = 3). (I) Schematic diagram of the lymph node immune assay is shown. C57BL/6 mice were subcutaneously inoculated with 2 × 10^5^ B16 melanoma cells and treated with daily intraperitoneal injections of fangchinoline (10 mg/kg) from day 2 to day 15. Tumor‐draining lymph nodes were collected on day 15 for downstream analysis. (J,K) Mice treated with fangchinoline (10 mg/kg, intraperitoneally) for 14 days were sacrificed, and tumor‐draining lymph nodes were harvested, digested, and analyzed by flow cytometry. The proportions of cDC1 cells among immune cells and H‐2K^b^–SIINFEKL^+^ cDC1 cells among total cDC1 cells were quantified. Analyzed by two‐tailed unpaired *t*‐test (n = 4). Fan, fangchinoline; MFI, mean fluorescence intensity. *p < 0.05; ** *p* < 0.01; ***p < 0.001; ns: not significant.

To identify small molecules that could restore or enhance DC cross‐presentation, we established a cell‐based in vitro screening system using ovalbumin (OVA) as a model antigen [31]. A natural product library comprising ∼1500 compounds (TargetMol L6000) was screened for agents that enhance MHC‐I–restricted presentation of OVA‐derived peptides. Fangchinoline emerged as a top hit, significantly enhancing OVA cross‐presentation as measured by H‐2K^b^–SIINFEKL surface expression (Figure 1A). The chemical structure of fangchinoline is shown in Figure 1B, and its low cytotoxicity to MutuDCs was confirmed by viability assays (Figure S1B).

Subsequent validation experiments showed that fangchinoline enhanced cross‐presentation of full‐length OVA in both MutuDCs and primary bone marrow–derived DCs, including GM‐CSF/IL‐4–differentiated BMDCs (G‐BMDCs) and Flt3L‐induced BMDCs (F‐BMDCs) (Figure 1C–E; Figure S1C). In the absence of full‐length OVA, however, fangchinoline did not affect H‐2K^b^–SIINFEKL levels in MutuDCs (Figure S1D).

Furthermore, bead uptake assays demonstrated that fangchinoline did not alter the phagocytic capacity of MutuDCs (Figure S1E). Notably, fangchinoline also had no effect on direct peptide presentation, as treatment with fangchinoline did not alter the presentation of the OVA (257–264) peptide (SIINFEKL), indicating that its effect is specific to the antigen processing and cross‐presentation pathway (Figure S1F).

To further determine whether fangchinoline influenced the expression of antigen‐presenting and co‐stimulatory molecules, we analyzed levels of MHC‐I (H‐2K^b^), MHC‐II (I‐A/I‐E), CD80, and CD86. Fangchinoline did not alter basal MHC‐I expression (Figure S1G) and did not induce upregulation of MHC‐II, CD80, or CD86, in contrast to the TLR9 agonist CpG, which served as a positive control (Figure S1H,I). In addition, fangchinoline failed to stimulate secretion of the proinflammatory cytokine IL‐12/23p40 (Figure S1J). These results suggest that fangchinoline enhances cross‐presentation without inducing MutuDCs maturation or inflammatory activation.

To evaluate the functional consequences of enhanced cross‐presentation, we next assessed OT‐I CD8^+^ T cell activation in co‐culture with fangchinoline‐treated MutuDCs. MutuDCs were pulsed with full‐length OVA in the presence or absence of fangchinoline and co‐cultured with OT‐I T cells. Fangchinoline treatment led to significantly increased secretion of IFN‐γ by OT‐I cells (Figure 1F,G), as well as enhanced T cell–mediated cytotoxicity against B16‐OVA cells, as measured by apoptosis induction in target cells (Figure 1H; gating strategy shown in Figure S2A).

To investigate the in vivo relevance of these findings, we established a B16‐OVA tumor model in C57BL/6 mice. Daily intraperitoneal administration of fangchinoline (10 mg/kg) was initiated on day 2 post tumor inoculation. On day 15, tumor‐draining lymph nodes were analyzed by flow cytometry. Fangchinoline‐treated mice showed no significant change in the number of conventional type 1 dendritic cells (cDC1s). However, the proportion of H‐2K^b^–SIINFEKL^+^ cDC1s was increased (Figure 1I–K; gating strategy shown in Figure S2B), indicating that Fangchinoline does not affect the abundance of cDC1s in vivo, but still enhances the antigen cross‐presentation capacity of DCs.

Together, these data support that fangchinoline selectively enhances dendritic cell antigen cross‐presentation, rather than broadly increasing antigen uptake, peptide presentation, or canonical maturation/inflammatory activation. Consistent with this, fangchinoline promotes antigen‐specific CD8^+^ T‐cell responses in vitro and in vivo. These findings support further investigation of fangchinoline as a small‐molecule modulator of DC‐mediated immunity.

### 2.2 Fangchinoline Activates DC‐Mediated Anti‐Tumor Immunity in Melanoma Models via CD8^+^ T Cell Responses

**FIGURE 2:**
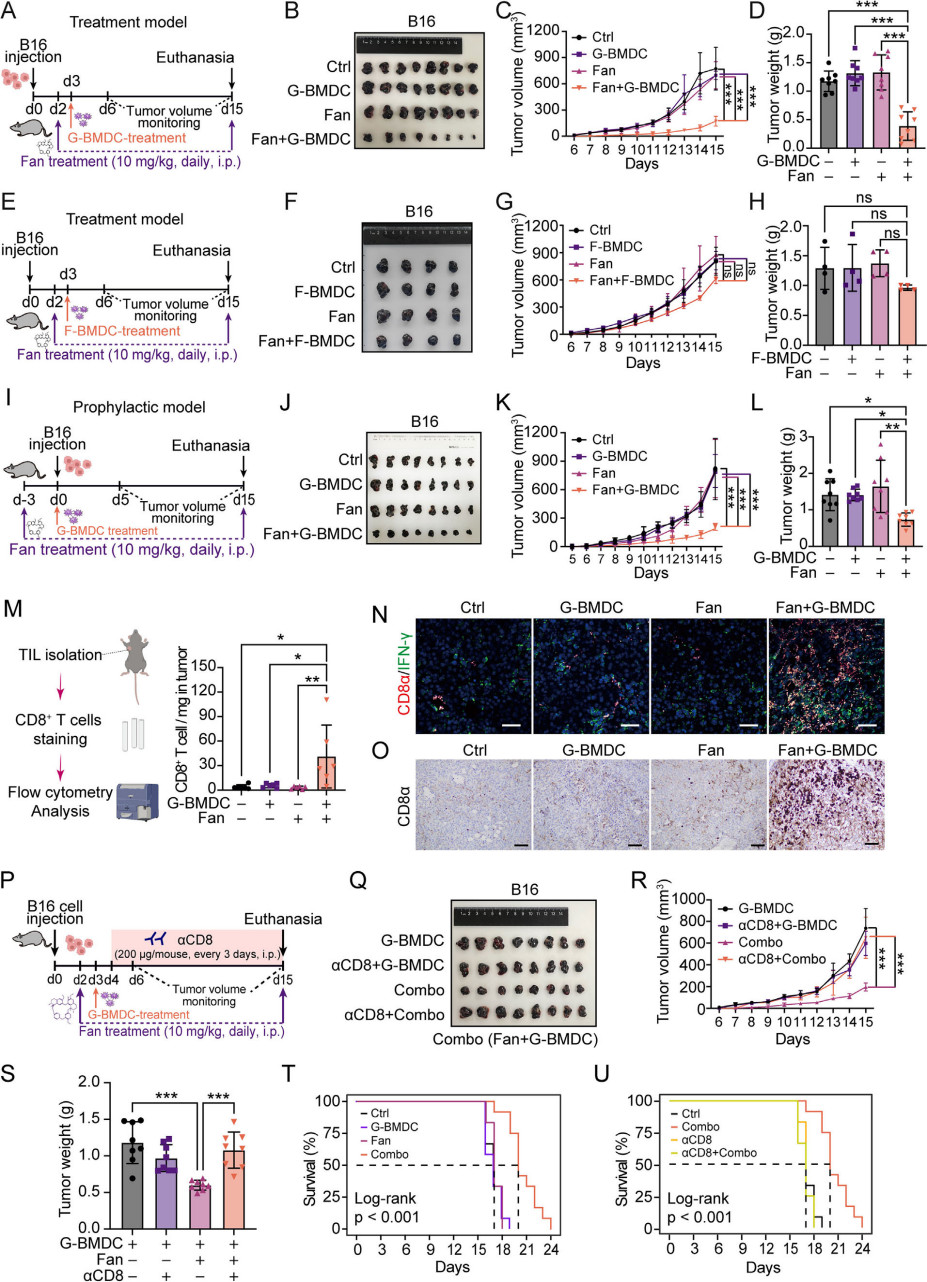
Fangchinoline activates DC‐mediated anti‐tumor immunity in melanoma models via CD8^+^ T cell responses. (A) Schematic of the G‐BMDC treatment model. C57BL/6 mice were subcutaneously inoculated in the axilla with 2 × 10^5^ B16 melanoma cells. On day 3, 5 × 10^4^ G‐BMDCs were injected at the tumor site. Fangchinoline (10 mg/kg/day) was administered intraperitoneally from day 2 to day 15. Experimental groups included: Control, G‐BMDC, fangchinoline, and fangchinoline + G‐BMDC. Tumors were harvested on day 15. (B–D) Representative tumor images, tumor growth curves, and tumor weights from the G‐BMDC model. Data were analyzed by two‐way ANOVA followed by Tukey's post hoc test (n = 8). (E) Schematic of the F‐BMDC treatment model. C57BL/6 mice were inoculated subcutaneously with 2 × 10^5^ B16 cells. On day 3, 5 × 10^4^ Flt3L‐induced BMDCs (F‐BMDCs) were administered at the tumor site. Fangchinoline (10 mg/kg/day) was injected intraperitoneally from day 2 to day 15. Groups included: Control, F‐BMDC, fangchinoline, and fangchinoline + F‐BMDC. Tumors were collected on day 15. (F–H) Tumor images, growth curves, and tumor weights from the F‐BMDC model. Data were analyzed by two‐way ANOVA followed by Tukey's post hoc test (n = 4). (I) Schematic of the prophylactic model. C57BL/6 mice were inoculated with a mixture of 2 × 10^5^ B16 cells and 5 × 10^4^ G‐BMDCs. Fangchinoline (10 mg/kg/day) was administered intraperitoneally from day −3 to day 15. Groups included: Control, G‐BMDC, fangchinoline, and fangchinoline + G‐BMDC. Tumors were harvested on day 15. (J–L) Representative tumor images, tumor growth curves, and tumor weights from the prophylactic model. Data were analyzed by two‐way ANOVA followed by Tukey's post hoc test (n = 8). (M) Flow cytometric analysis of CD8^+^ T cell infiltration in tumor tissues from indicated groups: Control, G‐BMDC, fangchinoline, and fangchinoline + G‐BMDC. Data were analyzed by two‐way ANOVA followed by Tukey's post hoc test (n = 6). (N) Immunofluorescence staining of B16 tumor sections for CD8α (red) and IFN‐γ (green). Nuclei were counterstained with Hoechst 33342. Scale bars, 50 µm. (O) Immunohistochemical staining of CD8α^+^ T cells in tumor sections. Areas of CD8α positivity are shown in brown. Scale bar, 100 µm. (P) Schematic of the anti‐CD8α depletion model. C57BL/6 mice were inoculated with 2 × 10^5^ B16 cells and 5 × 10^4^ G‐BMDCs, and treated with fangchinoline (10 mg/kg/day, intraperitoneally) from day 2 to day 15. Anti‐CD8α antibody (200 µg/mouse) was administered every 3 days starting on day 4. (Q–S) Representative tumor images, tumor growth curves, and tumor weights from the anti‐CD8α reversal model. Data were analyzed by two‐way ANOVA followed by Tukey's post hoc test (n = 8). (T) Kaplan–Meier survival analysis of tumor‐bearing mice treated with fangchinoline and/or G‐BMDC. Combination treatment significantly prolonged survival compared to controls. Statistical analysis by log‐rank (Mantel–Cox) test (n  =  12). (U) Kaplan–Meier survival analysis showing that CD8α depletion abrogated the survival benefit of fangchinoline + G‐BMDC therapy. Groups: Control, fangchinoline + G‐BMDC, anti‐CD8α, and fangchinoline + G‐BMDC + anti‐CD8α. Statistical analysis by log‐rank test (n = 12). Fan, fangchinoline. *p < 0.05; ** *p* < 0.01; ***p < 0.001; ns: not significant.

To systematically evaluate the in vivo anti‐tumor efficacy of fangchinoline and its synergy with dendritic cells (DCs), we employed murine melanoma models and assessed various DC subsets under both therapeutic and prophylactic conditions. In the B16 therapeutic model, bone marrow–derived DCs generated using granulocyte‐macrophage colony‐stimulating factor (GM‐CSF) and interleukin‐4 (IL‐4)—referred to as G‐BMDCs—were used as a classical in vitro DC vaccine [32]. While neither fangchinoline nor G‐BMDCs alone significantly suppressed tumor growth, their combination elicited marked tumor inhibition (Figure 2A–D).

Given the pivotal role of conventional type 1 DCs (cDC1s) in cross‐priming cytotoxic CD8^+^ T cells within the tumor microenvironment [28, 33], we also tested Flt3L‐induced BMDCs (F‐BMDCs), which are enriched in cDC1‐like phenotypes. However, the combination of fangchinoline with F‐BMDCs conferred only modest therapeutic benefit, and neither treatment alone was effective (Figure 2E–H). To exclude the possibility that these results were confounded by batch‐to‐batch variation in the cellular composition of in vitro–generated F‐BMDCs, we assessed the transfer inocula in parallel for the frequency of XCR1^+^ cells, which represent the cDC1‐enriched fraction in these cultures (Figure S3A). The limited efficacy of fangchinoline monotherapy likely reflects the insufficient accumulation of endogenous DCs at the tumor site [34], while the poor response to DC monotherapy may result from the immunosuppressive tumor milieu that impairs DC functionality and T cell activation [29, 35, 36].

These findings were further validated in a prophylactic setting. Co‐inoculation of B16 cells with G‐BMDCs, followed by fangchinoline administration, significantly delayed tumor onset and reduced tumor burden compared to individual treatments (Figure 2I–L). Similar results were observed in a B16‐OVA prophylactic model, where G‐BMDC treatment alone conferred partial protection, which was further enhanced by fangchinoline co‐administration (Figure S3B–E).

To further probe the role of DC abundance in therapeutic efficacy, we generated a B16 melanoma cell line stably overexpressing Flt3L (B16‐OE‐Flt3L), a key cytokine required for cDC1 development [37]. Flt3L overexpression and function were confirmed by qPCR and tumor‐conditioned medium (TCM)–based assays, which showed increased proportions of XCR1^+^ BMDCs in vitro (Figure S3F–H). In vivo, B16‐OE‐Flt3L tumors exhibited slower growth than wild‐type B16 tumors, suggesting that elevated Flt3L expression may partially enhance anti‐tumor immunity (Figure S3I–L). However, additional fangchinoline administration in this context did not further improve tumor control, indicating limited synergistic benefit in tumors with high endogenous Flt3L expression (Figure S3I–L). These findings suggest that combining fangchinoline with F‐BMDCs may not represent an optimal therapeutic strategy. The mechanistic basis underlying the differential efficacy between fangchinoline + F‐BMDCs and fangchinoline + G‐BMDCs was further investigated and is detailed in the following section. Based on these observations, G‐BMDCs were selected as the preferred partner for fangchinoline‐based combination therapy. This also aligns with previous studies, as GM‐CSF + IL‐4–induced DCs are widely recognized as a standard platform for DC vaccine development [38, 39].

To elucidate the underlying mechanism, we examined the tumor immune microenvironment. Flow cytometric analysis revealed a significant increase in the infiltration of CD8^+^ T cells following combination therapy (Figure 2M). Immunofluorescence staining showed enhanced colocalization of CD8α^+^ T cells with effector molecules IFN‐γ and granzyme B (GZMB), indicating a functionally active cytotoxic response (Figure 2N; Figure S3M). Immunohistochemistry further confirmed a higher density of CD8α^+^ T cells in the combination group compared to controls (Figure 2O).

To assess the functional importance of CD8^+^ T cells in mediating the therapeutic effect, we performed in vivo depletion using anti‐CD8α antibodies. Elimination of CD8^+^ T cells completely abrogated the tumor‐suppressive effect of fangchinoline + G‐BMDC therapy (Figure 2P–S) and significantly diminished the associated survival benefit (Figure 2T–U). Together, these data demonstrate that CD8^+^ T cells are indispensable for the anti‐tumor activity of fangchinoline and G‐BMDCs, acting as the principal effector population responsible for tumor control.

### 2.3 DNGR‐1 Is Required for Fangchinoline‐Boosted Dendritic Cell Cross‐Presentation and CD8^+^ T Cell Priming In Vitro

**FIGURE 3:**
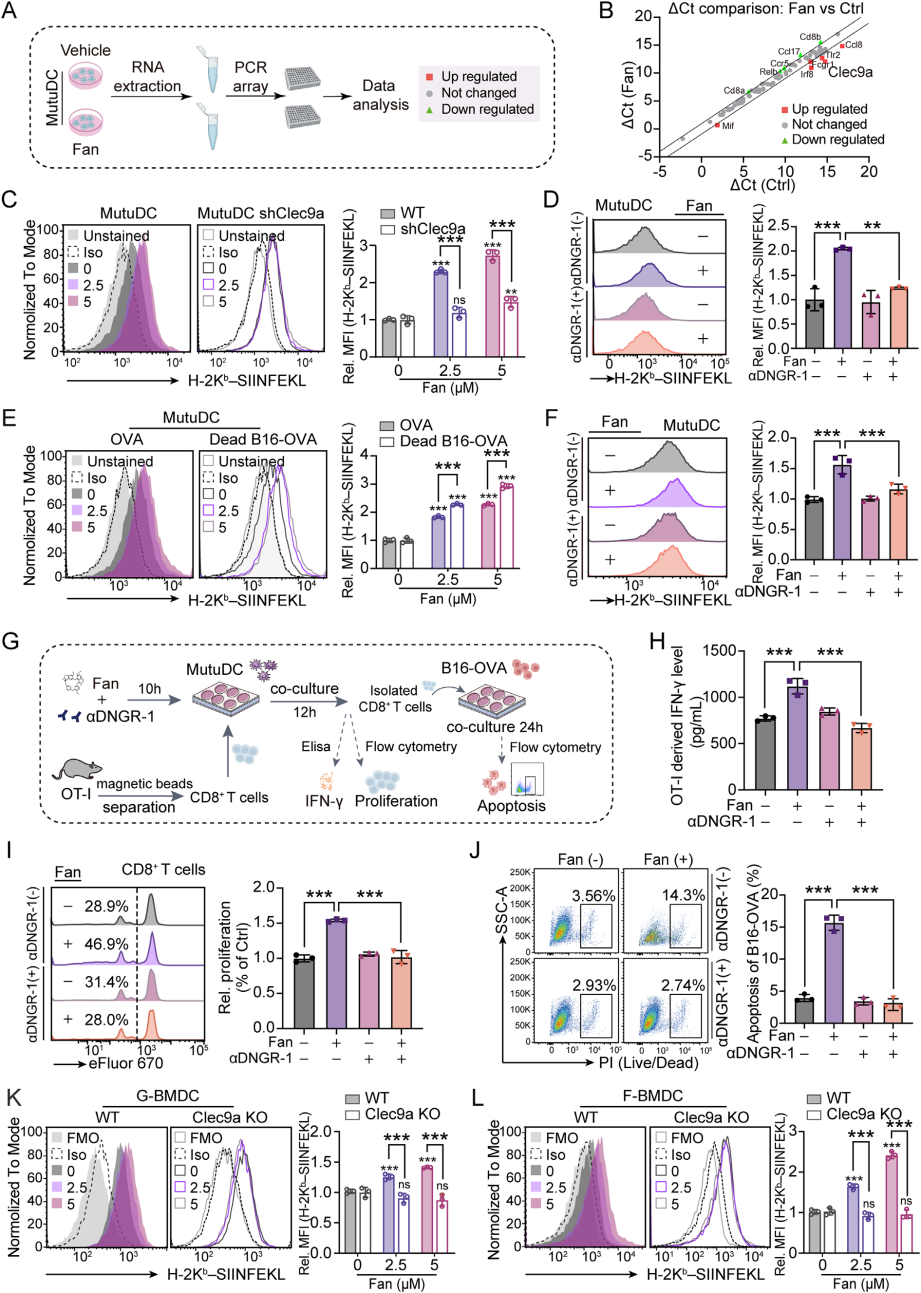
DNGR‐1 is required for fangchinoline‐boosted dendritic cell cross‐presentation and CD8^+^ T cell priming in vitro. (A) Schematic of PCR array screening. MutuDCs were treated with fangchinoline, followed by RNA extraction and gene expression profiling. (B) PCR array identified Clec9a, a gene closely associated with dendritic cell antigen cross‐presentation, as significantly upregulated upon fangchinoline treatment. (C) Flow cytometric analysis of antigen cross‐presentation in OVA‐loaded wild‐type (WT) and Clec9a knockdown (shClec9a) MutuDCs treated with fangchinoline (2.5 or 5 µM). The MFI of cell‐surface H‐2K^b^–SIINFEKL complexes was reduced in shClec9a cells. Data analyzed by two‐way ANOVA with Tukey's post hoc test (n = 3). (D) In OVA‐loaded MutuDCs, fangchinoline (5 µM), either alone or in combination with anti‐DNGR‐1 antibody (αDNGR‐1), altered the MFI of cell‐surface H‐2K^b^–SIINFEKL complexes, as measured by flow cytometry. Data analyzed by two‐way ANOVA with Tukey's post hoc test (n = 3). (E) MutuDCs were stimulated with B16‐OVA dead cell antigen or soluble OVA (200 µg/mL) in the presence of fangchinoline (2.5 or 5 µM) for 24 h. H‐2K^b^–SIINFEKL MFI was assessed by flow cytometry and normalized to control. Data analyzed by two‐way ANOVA with Tukey's post hoc test (n = 3). (F) MutuDCs loaded with B16‐OVA dead cell antigen were treated with fangchinoline (5 µM) alone or in combination with anti‐DNGR‐1 antibody (αDNGR‐1). The MFI of cell‐surface H‐2K^b^–SIINFEKL complexes was measured by flow cytometry. Data were analyzed by two‐way ANOVA with Tukey's post hoc test (n  =  3). (G) Schematic of the in vitro cytotoxicity assay. MutuDCs were treated with fangchinoline and/or αDNGR‐1, followed by co‐culture with OT‐I CD8^+^ T cells and B16‐OVA target cells. T cell function was assessed via IFN‐γ secretion, proliferation, and cytotoxicity. (H) IFN‐γ levels in the supernatant were measured by ELISA following co‐culture of OT‐I T cells with fangchinoline‐ and/or αDNGR‐1–treated MutuDCs. Data analyzed by two‐way ANOVA with Tukey's post hoc test (n = 3). (I) OT‐I T cell proliferation was assessed after co‐culture with pretreated MutuDCs using eFluor 670 dye dilution. Data analyzed by two‐way ANOVA with Tukey's post hoc test (n = 3). (J) Flow cytometric analysis of B16‐OVA cell apoptosis after 24‐h co‐culture with OT‐I CD8^+^ T cells that were pre‐activated by fangchinoline‐ and/or αDNGR‐1–treated MutuDCs. Apoptotic B16‐OVA cells were quantified. Data analyzed by two‐way ANOVA with Tukey's post hoc test (n = 3). (K) Flow cytometric analysis of antigen cross‐presentation in OVA‐loaded WT and Clec9a knockout (KO) G‐BMDCs treated with fangchinoline (2.5 or 5 µM). The MFI of cell‐surface H‐2K^b^–SIINFEKL complexes was significantly reduced in KO cells. Data analyzed by two‐way ANOVA with Tukey's post hoc test (n = 3). (L) Similar analysis in OVA‐loaded WT and Clec9a KO F‐BMDCs treated with fangchinoline. No significant increase in the MFI of cell‐surface H‐2K^b^–SIINFEKL complexes was observed in KO cells. Data analyzed by two‐way ANOVA with Tukey's post hoc test (n = 3). Fan, fangchinoline; MFI, mean fluorescence intensity; G‐BMDC, GM‐CSF/IL‐4–induced bone marrow–derived dendritic cells; F‐BMDC, Flt3L‐induced bone marrow–derived dendritic cells; αDNGR‐1, anti‐DNGR‐1 neutralizing antibody; WT, wild‐type; KO, knockout. *p < 0.05; ** *p* < 0.01; ***p < 0.001; ns: not significant.

To elucidate the molecular mechanism by which fangchinoline enhances antigen cross‐presentation in dendritic cells, we performed PCR array screening to identify differentially expressed genes in fangchinoline‐treated MutuDCs. Among the upregulated genes, Clec9a—encoding DNGR‐1, a receptor closely associated with cross‐presentation—was significantly induced upon treatment (Figure 3A,B; a detailed list of differentially expressed genes is provided in Table S2).

To validate the functional relevance of DNGR‐1, we generated stable knockdown MutuDC cell lines using shRNA targeting Clec9a. Knockdown efficiency was confirmed by qPCR and western blot (Figure S4A,B). In parallel, a neutralizing antibody against DNGR‐1 was used to block receptor activity. Both genetic silencing and antibody‐mediated inhibition of DNGR‐1 significantly impaired fangchinoline‐induced enhancement of antigen cross‐presentation, as indicated by reduced H‐2K^b^–SIINFEKL levels in OVA‐loaded dendritic cells (Figure 3C,D).

To further validate the functional relevance of DNGR‐1 in a more physiologically relevant antigen context, we used dead B16 and B16‐OVA cells as sources of cell‐associated antigens, in parallel with soluble full‐length OVA. Fangchinoline enhanced the cross‐presentation of dead‐cell–associated antigens derived from B16‐OVA cells to a greater extent than in the soluble OVA setting (Figure 3E), but had no appreciable effect on the cross‐presentation of antigens derived from dead OVA‐negative B16 cells (Figure S4D). Notably, this enhancement was substantially diminished upon DNGR‐1/CLEC9A interference (Figure 3F), supporting a DNGR‐1‐dependent effect of fangchinoline in a dead cell–associated antigen setting that is closely aligned with the canonical biology of DNGR‐1.

We next assessed the impact of DNGR‐1 inhibition on fangchinoline‐mediated activation of CD8^+^ T cells. In an in vitro cytotoxicity assay, dendritic cells pretreated with fangchinoline and/or anti‐DNGR‐1 antibody were co‐cultured with OT‐I CD8^+^ T cells and B16‐OVA target cells (Figure 3G). Blockade of DNGR‐1 significantly reduced IFN‐γ secretion by OT‐I T cells (Figure 3H), impaired T cell proliferation as assessed by eFluor 670 dye dilution (Figure 3I), and diminished tumor cell killing, as indicated by lower apoptosis rates in B16‐OVA cells (Figure 3J; gating strategy shown in Figure S4C). These data collectively support that DNGR‐1 activity is critical for fangchinoline‐induced dendritic cell–mediated CD8^+^ T cell priming and cytotoxic function.

To further confirm these findings in a genetic knockout model, we generated bone marrow–derived DCs (BMDCs) from wild‐type and Clec9a knockout (KO) C57BL/6J mice. Using GM‐CSF/IL‐4 or Flt3L, cells were differentiated into G‐BMDCs and F‐BMDCs, respectively. Successful deletion of DNGR‐1 was validated by flow cytometry (Figure S4E). In both BMDC types, loss of DNGR‐1 abolished the fangchinoline‐induced increase in antigen cross‐presentation, as evidenced by the absence of H‐2K^b^–SIINFEKL induction in Clec9a KO cultures (Figure 3K,L). These results indicate that DNGR‐1 is required for fangchinoline‐enhanced cross‐presentation in the DC systems tested.

Together, these results support that fangchinoline enhances dendritic cell–mediated antigen cross‐presentation and subsequent CD8^+^ T cell activation through a DNGR‐1–dependent mechanism.

### 2.4 DNGR‐1 Is Required for Fangchinoline‐Boosted Dendritic Cell Antitumor Activity In Vivo

**FIGURE 4:**
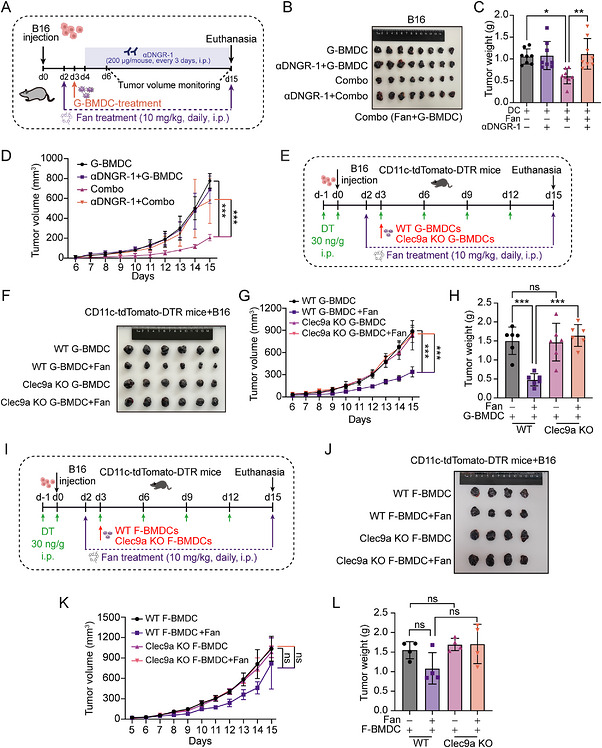
DNGR‐1 is required for fangchinoline‐boosted dendritic cell antitumor activity in vivo. (A) Schematic of the experimental timeline. C57BL/6 mice were subcutaneously inoculated with 2 × 10^5^ B16 melanoma cells on day 0. Starting on day 2, mice received daily intraperitoneal injections of fangchinoline (10 mg/kg). On day 3, G‐BMDCs were administered, and anti‐DNGR‐1 (αDNGR‐1; 200 µg per mouse) was injected intraperitoneally every 3 days starting on day 4. (B–D) Tumors were collected on day 15. Representative tumor images, tumor weights, and tumor growth curves are shown. Data analyzed by two‐way ANOVA with Tukey's post hoc test (n = 8). (E) Schematic of the dendritic cell–specific ablation model. CD11c‐tdTomato‐DTR mice were inoculated with 2 × 10^5^ B16 cells on day 0. Diphtheria toxin (DT, 30 ng/g) was administered on days −1, 0, and every 3 days thereafter to deplete endogenous CD11c^+^ cells. On day 3, 5 × 10^4^ G‐BMDCs from either wild‐type (WT) or Clec9a knockout (KO) mice were injected. Mice received daily fangchinoline (10 mg/kg) intraperitoneally from day 1 to day 15. Tumors were harvested on day 15. (F–H) Representative tumor images, tumor volumes, and weights from the G‐BMDC reconstitution experiment. Data analyzed by two‐way ANOVA with Tukey's post hoc test (n = 6). (I) Schematic of the F‐BMDC reconstitution model. CD11c‐tdTomato‐DTR mice were treated as in (E), except that 5 × 10^4^ F‐BMDCs from WT or Clec9a KO mice were transferred on day 3. Fangchinoline was administered daily as described. (J–L) Representative tumor images, tumor volumes, and tumor weights from the F‐BMDC reconstitution experiment. Data analyzed by two‐way ANOVA with Tukey's post hoc test (n = 4). Fan, fangchinoline; G‐BMDC, GM‐CSF/IL‐4–induced bone marrow–derived dendritic cells; F‐BMDC, Flt3L‐induced bone marrow–derived dendritic cells; αDNGR‐1, anti‐DNGR‐1 neutralizing antibody; WT, wild‐type; KO, knockout; ns, not significant. *p < 0.05; ** *p* < 0.01; ***p < 0.001; ns: not significant.

To investigate the role of DNGR‐1 in the antitumor efficacy of fangchinoline, we first established a subcutaneous melanoma model. C57BL/6 mice bearing B16 tumors were treated with fangchinoline in combination with GM‐CSF/IL‐4–induced bone marrow–derived dendritic cells (G‐BMDCs), with or without concurrent administration of a neutralizing anti‐DNGR‐1 antibody. Blockade of DNGR‐1 significantly diminished the tumor growth inhibition observed with fangchinoline and G‐BMDC co‐treatment, as evidenced by increased tumor size and weight (Figure 4A–D). These results suggest that DNGR‐1 is required for the therapeutic synergy between fangchinoline and dendritic cell–based immunotherapy.

To further dissect the DC‐intrinsic role of DNGR‐1, we employed a conditional DC depletion model using CD11c‐tdTomato‐DTR mice. Administration of diphtheria toxin (DT) selectively ablated endogenous CD11c^+^ dendritic cells, as confirmed by flow cytometric analysis of tdTomato^+^ and CD11c^+^ populations (Figure S4F). In this DC‐ablated setting, we adoptively transferred G‐BMDCs from either wild‐type (WT) or Clec9a knockout (KO) mice, in combination with daily fangchinoline administration. While WT G‐BMDCs synergized with fangchinoline to suppress tumor growth, this effect was substantially reduced when Clec9a‐deficient G‐BMDCs were used (Figure 4E–H), confirming a cell‐intrinsic requirement for DNGR‐1 in DCs.

To determine whether the observed synergy was solely attributable to DNGR‐1 expression levels, we performed a parallel experiment using Flt3L‐induced BMDCs (F‐BMDCs), which exhibit high baseline DNGR‐1 expression. In parallel, the F‐BMDC preparations used for adoptive transfer were evaluated for the frequency of XCR1^+^ cells, which represent the cDC1‐like subset in these cultures (Figure S4G). In the same DC‐ablated model, neither wild‐type nor *Clec9a* knockout F‐BMDCs conferred significant tumor control when combined with fangchinoline (Figure 4I–L). These findings suggest that the efficacy of fangchinoline‐augmented DC therapy is not determined simply by the presence or abundance of DNGR‐1, but rather by a complex interplay between DNGR‐1 regulation and the cellular context of distinct DC subsets. The differential response between G‐BMDCs and F‐BMDCs likely reflects their unique transcriptional or functional programs, which we further characterize in the following section.

### 2.5 Fangchinoline Enhances DNGR‐1 Expression in G‐BMDCs but Induces Apoptosis in DNGR‐1–High F‐BMDCs

**FIGURE 5:**
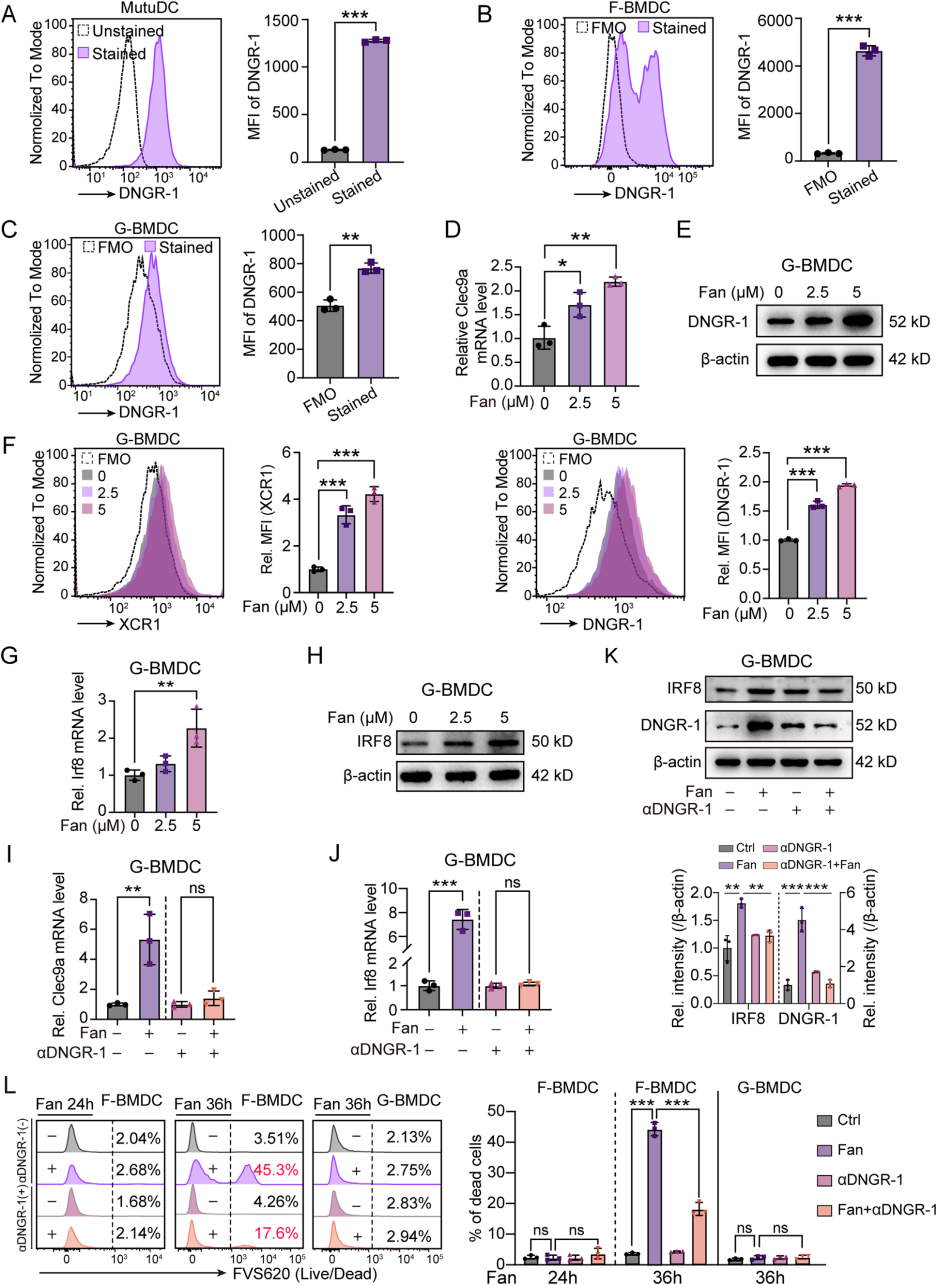
Fangchinoline upregulates DNGR‐1 in GM‐CSF–derived dendritic cells. (A–C) Flow cytometric analysis of baseline DNGR‐1 expression in CD11c^+^ dendritic cells from MutuDCs (A), Flt3L‐induced BMDCs (F‐BMDCs; B), and GM‐CSF/IL‐4–induced BMDCs (G‐BMDCs; C), using isotype control and anti‐DNGR‐1 staining. Representative flow cytometry plots show DNGR‐1 expression within the CD11c^+^ gate in all three dendritic cell subsets. Bar graphs depict the corresponding MFI values. Analyzed by two‐tailed unpaired t‐test (n = 3). (D) Clec9a mRNA expression in fangchinoline‐treated G‐BMDCs, measured by qPCR and normalized to Actb. Analyzed by one‐way ANOVA with Tukey's post hoc test (n = 3). (E) Western blot analysis of DNGR‐1 protein levels in G‐BMDCs treated with fangchinoline (0, 2.5, or 5 µM). (F) Representative flow cytometry plots show DNGR‐1 and XCR1 expression in CD11c^+^ G‐BMDCs after fangchinoline treatment. Graphs quantify the relative change in MFI of DNGR‐1 and XCR1 within the CD11c^+^ gate; relative MFI was calculated as fold change over the untreated control. Analyzed by one‐way ANOVA with Tukey's post hoc test (n = 3). (G) Irf8 mRNA expression in fangchinoline‐treated G‐BMDCs, assessed by qPCR. Analyzed by one‐way ANOVA with Tukey's post hoc test (n = 3). (H) Western blot analysis of IRF8 protein levels in G‐BMDCs following fangchinoline treatment. (I,J) Clec9a (I) and Irf8 (J) mRNA expression in G‐BMDCs treated with fangchinoline alone or in combination with anti‐DNGR‐1 (αDNGR‐1) antibody. Analyzed by two‐tailed unpaired t‐test (n = 3). (K) Western blot analysis of IRF8 and DNGR‐1 protein levels in G‐BMDCs treated with fangchinoline and/or αDNGR‐1. Data analyzed by two‐way ANOVA with Tukey's post hoc test (n = 3). (L) Apoptosis analysis of F‐BMDCs and G‐BMDCs treated with fangchinoline and/or αDNGR‐1 for 24 or 36 h using live/dead staining. Data analyzed by two‐way ANOVA with Tukey's post hoc test (n = 3). Fan, fangchinoline; G‐BMDC, GM‐CSF/IL‐4–induced bone marrow–derived dendritic cell; F‐BMDC, Flt3L‐induced bone marrow–derived dendritic cell; MFI, mean fluorescence intensity; αDNGR‐1, anti‐DNGR‐1 neutralizing antibody; *p < 0.05; ** *p* < 0.01; ***p < 0.001; ns: not significant.

Consistent with previous reports [40], GM‐CSF/IL‐4–induced bone marrow–derived dendritic cells (G‐BMDCs) exhibit relatively low baseline expression of DNGR‐1, in contrast to the high expression observed in MutuDCs and Flt3L‐induced BMDCs (F‐BMDCs) (Figure 5A–C). This observation prompted us to investigate how fangchinoline enhances the antigen cross‐presentation capacity of G‐BMDCs via DNGR‐1 and achieves a more potent antitumor response in vivo than F‐BMDCs (Figure 2A–H).

Initial PCR array screening identified Clec9a and Irf8 among the transcripts upregulated by fangchinoline in MutuDCs (Figure 3B), implicating a possible link to the cross‐presenting DC program. We therefore examined DNGR‐1/CLEC9A expression in additional DC systems. In GM‐CSF/IL‐4‐derived BMDCs, fangchinoline markedly increased DNGR‐1 expression at both the transcript and protein levels, as determined by qPCR and immunoblotting (Figure 5D–E). Consistently, flow cytometry showed elevated surface expression of DNGR‐1 and XCR1 in CD11c^+^ G‐BMDCs after fangchinoline treatment (Figure 5F). Further subset analysis of GM‐CSF/IL‐4‐derived cultures, using the gating strategy shown in Figure S5A, demonstrated that DNGR‐1 expression was increased in both the DC‐like and macrophage‐like compartments (Figure S5B,C). By contrast, in Flt3L‐derived DC cultures, analysis of cDC1 and cDC2 subsets (Figure S5D) showed no significant change in DNGR‐1 expression following fangchinoline treatment (Figure S5E,F). These results indicate that fangchinoline‐mediated regulation of DNGR‐1 is context dependent and is more evident in GM‐CSF/IL‐4‐derived DC cultures than in Flt3L‐derived DCs.

IRF8 is a key transcription factor governing dendritic cell lineage specification, particularly cDC1 development, and has been shown to regulate Clec9a expression, potentially through direct binding to its promoter or enhancer regions [15]. Consistent with this, fangchinoline robustly increased Irf8 mRNA and IRF8 protein expression in G‐BMDCs (Figure 5G,H), suggesting that IRF8 may mediate the transcriptional upregulation of DNGR‐1. To examine whether this process depends on DNGR‐1 signaling, we co‐treated G‐BMDCs with fangchinoline and a neutralizing anti‐DNGR‐1 antibody. DNGR‐1 blockade significantly reduced the fangchinoline‐induced expression of both *Irf8* and IRF8 protein (Figure 5I–K), indicating a potential positive feedback loop between DNGR‐1 activation and IRF8 expression.

These results provide mechanistic insight into how fangchinoline enhances selected cross‐presentation‐associated features of G‐BMDCs through IRF8‐associated upregulation of DNGR‐1, thereby supporting a more effective antitumor response in this system.

In contrast, prolonged exposure to fangchinoline (36 h) markedly increased apoptosis in F‐BMDCs but had no significant effect on G‐BMDC viability (Figure 5L). This pro‐apoptotic effect in F‐BMDCs was largely reversed by DNGR‐1 blockade, suggesting that high baseline DNGR‐1 expression may sensitize F‐BMDCs to fangchinoline‐induced cytotoxic stress. These findings are consistent with prior work showing that DNGR‐1 constrains Flt3L‐mediated antitumor immunity by limiting the function of tumor‐infiltrating cDC1s [41]. Based on this and previous reports that DNGR‐1 can activate NADPH oxidase 2 (NOX2) to generate reactive oxygen species (ROS) within phagosomes [12], we speculate that fangchinoline may trigger excessive ROS accumulation in DNGR‐1–high F‐BMDCs, leading to oxidative stress–induced apoptosis and impaired antitumor function.

In summary, our findings uncover a subset‐context‐dependent regulatory mechanism by which fangchinoline modulates dendritic cell function and viability. In G‐BMDCs, fangchinoline promotes IRF8‐associated DNGR‐1 expression and induces selected cDC1‐associated markers together with a cross‐presentation‐associated functional program. Conversely, in DNGR‐1–high F‐BMDCs, prolonged fangchinoline exposure induces DNGR‐1‐dependent apoptotic cell death, which may limit their suitability as cellular vaccines in this setting. These results highlight the importance of considering DC subset identity and DNGR‐1 expression dynamics when designing optimized dendritic cell–based immunotherapies.

### 2.6 Fangchinoline Binds a Functional Pocket on DNGR‐1 Formed by Trp209, Glu225, and Arg226 to Enhance Antigen Cross‐Presentation in Dendritic Cells

**FIGURE 6:**
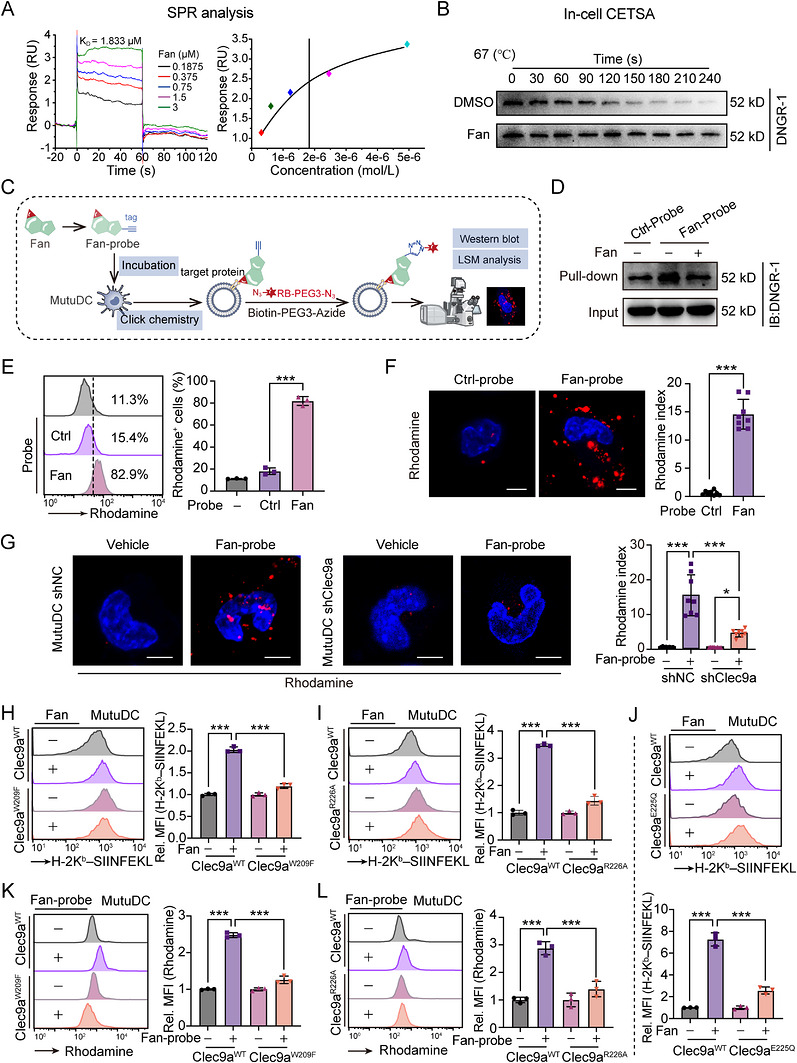
Fangchinoline binds DNGR‐1 at Trp209 and Arg226 to enhance antigen cross‐presentation in dendritic cells. (A) Surface plasmon resonance (SPR) analysis showing direct binding of fangchinoline to purified mouse DNGR‐1 immobilized on a CM5 sensor chip. Increasing concentrations of fangchinoline (0.1875–3 µM) produced a dose‐dependent response (K_D_ = 1.833 µM). (B) Cellular thermal shift assay (CETSA) assessing DNGR‐1 thermal stability in MutuDC membrane extracts treated with DMSO or 5 µM fangchinoline and heated at 67°C for the indicated times. DNGR‐1 stability was evaluated by immunoblotting. (C) Schematic of the Fan‐probe binding assay. Fangchinoline was conjugated to a clickable probe (Fan‐probe), incubated with MutuDCs, and labeled via click chemistry with biotin or fluorescent tags for detection by Western blot or confocal microscopy. (D) Pull‐down assay showing specific binding of Fan‐probe to DNGR‐1 in MutuDCs, competitively inhibited by excess free fangchinoline. Protein complexes were isolated using streptavidin Dynabeads and analyzed by immunoblotting. (E) Flow cytometry quantification of probe binding to MutuDCs treated with Fan‐probe or control probe (Ctrl‐probe, 5 µM). Analyzed by one‐way ANOVA with Tukey's post hoc test (n = 3). (F) Confocal microscopy images and quantification of rhodamine‐labeled probe binding in MutuDCs. Scale bars, 5 µm. Analyzed by two‐tailed unpaired t‐test (n = 8). (G) Confocal microscopy showing reduced Fan‐probe binding in Clec9a‐knockdown MutuDCs. Nuclei were stained with Hoechst 33342. Scale bars, 5 µm. Quantification by flow cytometry and microscopy. Data analyzed by two‐way ANOVA with Tukey's post hoc test (n = 8). (H–J) MutuDCs expressing wild‐type or Clec9aW209F or Clec9aE225Q or Clec9aR226A mutants were incubated with OVA (200 µg/mL) in the presence or absence of 5 µM fangchinoline. Antigen cross‐presentation was assessed by H‐2K^b^‐SIINFEKL staining via flow cytometry. Data analyzed by two‐way ANOVA with Tukey's post hoc test (n = 3). (K,L) Flow cytometry analysis of Fan‐probe binding in WT and Clec9a mutant MutuDCs (W209F and R226A), showing reduced fluorescence intensity. Data analyzed by two‐way ANOVA with Tukey's post hoc test (n = 3). Fan, fangchinoline; Ctrl‐probe, control probe; MFI, mean fluorescence intensity; *p < 0.05; ** *p* < 0.01; ***p < 0.001; ns: not significant.

We next investigated the molecular basis of fangchinoline–DNGR‐1 interaction and its functional relevance in dendritic cells. Surface plasmon resonance (SPR) analysis confirmed that fangchinoline directly binds to purified mouse DNGR‐1 with a dissociation constant (*K_D_*) of 1.833 µM, indicating a high‐affinity interaction (Figure 6A). Cellular thermal shift assays (CETSA) further demonstrated enhanced thermal stability of DNGR‐1 in the presence of fangchinoline, supporting a direct ligand–receptor interaction in a membrane context (Figure 6B).

To validate this interaction in cells, we synthesized a clickable fangchinoline‐derived probe (Fan‐probe), as illustrated in Figure S6A,B. Upon incubation with MutuDCs, Fan‐probe‐labeled proteins were biotinylated via click chemistry and captured using streptavidin‐coated magnetic beads. Immunoblot analysis revealed specific enrichment of DNGR‐1, which was competitively diminished by excess free fangchinoline, supporting binding specificity (Figure 6C,D). Flow cytometry analysis using a rhodamine‐labeled version of Fan‐probe showed robust membrane‐localized fluorescence in MutuDCs, whereas a control probe (Ctrl‐probe) showed negligible binding (Figure 6E; Figure S6C). Confocal microscopy further confirmed surface‐associated probe staining, and quantitative analysis revealed significantly higher signal intensity with Fan‐probe compared to control (Figure 6F). Notably, DNGR‐1 knockdown in MutuDCs markedly reduced Fan‐probe binding, as assessed by both microscopy and flow cytometry (Figure 6G), indicating that DNGR‐1 is the primary cellular target of fangchinoline.

To delineate the binding interface between fangchinoline and DNGR‐1, we conducted molecular docking simulations using the crystal structure of DNGR‐1 (PDB: 3J82). Among the predicted binding poses, the most energetically favorable configuration (−9.25 kcal/mol) revealed a pocket composed of Trp209, Glu225, and Arg226, where fangchinoline established multiple hydrogen bonds at distances of 2.2–2.6 A (Figure S6D). Additional potential contact residues included Arg154, Tyr252, and Lys258, but these interactions were associated with higher binding energies (−6.74 to −7.59 kcal/mol) and fewer hydrogen bonds.

To experimentally validate these predictions, we performed site‐directed mutagenesis of candidate residues in DNGR‐1 expressed in MutuDCs. Mutations at Trp209 (W209F), Glu225 (E225Q), and Arg226 (R226A) significantly impaired fangchinoline‐induced enhancement of antigen cross‐presentation, as assessed by surface expression of H‐2K^b^–SIINFEKL complexes following ovalbumin (OVA) uptake (Figure 6H–J). In contrast, mutations at Tyr252, Arg154, and Lys258 had minimal effects (Figure S6E). Flow cytometry analysis further demonstrated that Fan‐probe binding was markedly reduced in W209F and R226A mutant cells compared to wild‐type controls (Figure 6K,L), indicating that these residues are critical for ligand engagement.

Together, these results define a functional binding pocket on DNGR‐1—comprising Trp209, Glu225, and Arg226—that mediates fangchinoline recognition and contributes to the enhancement of antigen cross‐presentation in dendritic cells.

### 2.7 Fangchinoline Promotes Antigen Cross‐Presentation via DNGR‐1–Syk–Nox2–ROS–Mediated Phagosomal Permeabilization in Dendritic Cells

**FIGURE 7:**
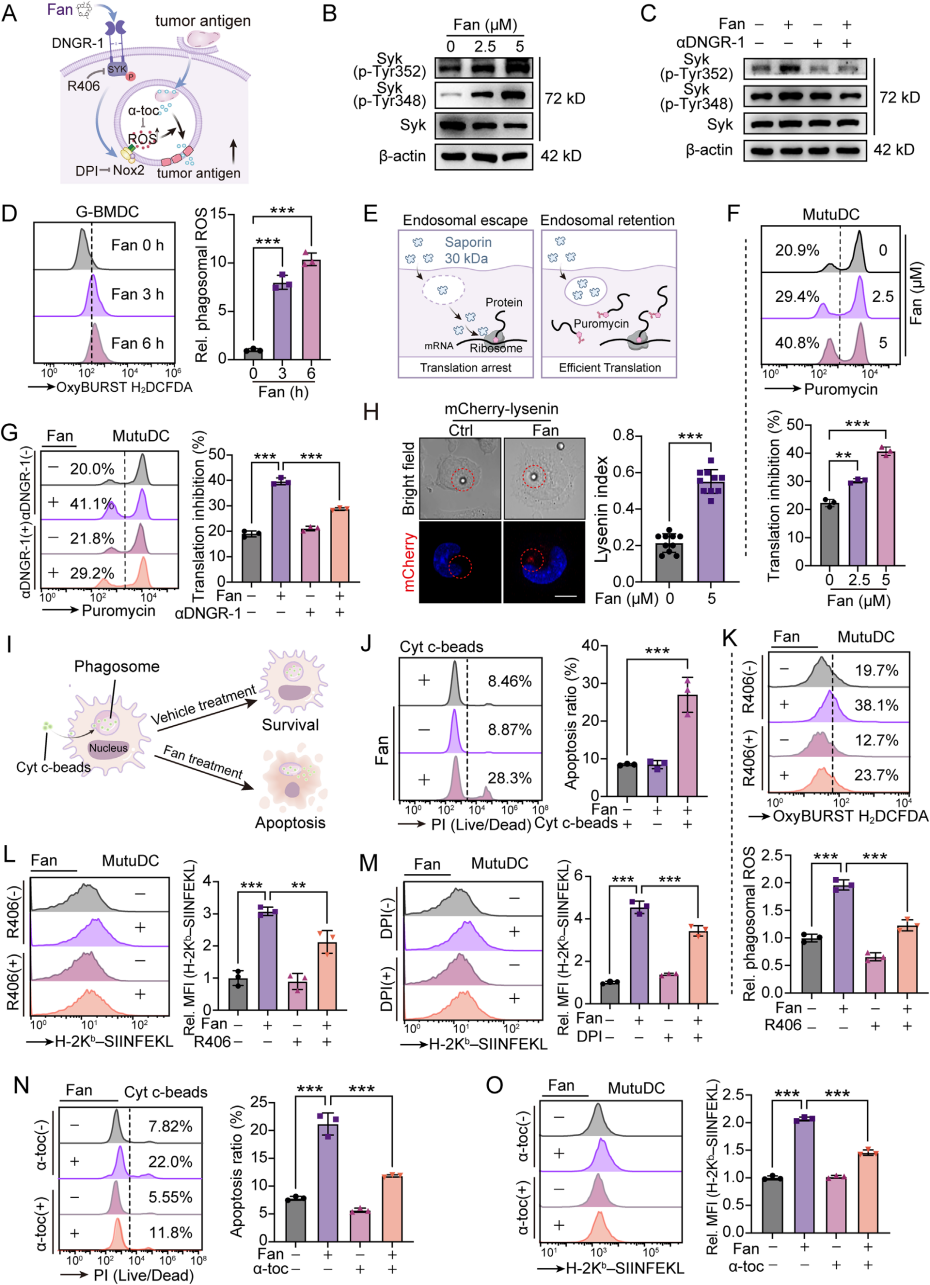
Fangchinoline enhances dendritic cell cross‐presentation by increasing phagosomal permeability via DNGR‐1–Syk–Nox2–ROS signaling. (A) Schematic of the proposed mechanism showing how fangchinoline promotes antigen cross‐presentation through DNGR‐1–mediated activation of the Syk–Nox2–ROS pathway. Key inhibitors used in subsequent experiments are also indicated. (B) Immunoblot analysis of Syk phosphorylation at Tyr348 and Tyr352 in MutuDCs treated with fangchinoline (2.5 or 5 µM) for 24 h, demonstrating dose‐dependent activation of Syk. (C) Syk phosphorylation induced by fangchinoline (5 µM) was markedly reduced upon DNGR‐1 blockade with a neutralizing antibody, confirming DNGR‐1‐dependent activation. (D) Phagosomal ROS accumulation in G‐BMDCs treated with fangchinoline (5 µM) for 3 or 6 h, quantified by flow cytometry using OxyBURST Green H_2_DCFDA–labeled amino microspheres. Analyzed by one‐way ANOVA with Tukey's post hoc test (n = 3). (E) Schematic illustration of the saporin–puromycin assay used to evaluate endosome‐to‐cytosol antigen escape. (F) Fangchinoline enhanced endosome‐to‐cytosol escape in MutuDCs, as determined by the saporin–puromycin assay. Analyzed by one‐way ANOVA with Tukey's post hoc test (n = 3). (G) The fangchinoline‐induced enhancement of endosome‐to‐cytosol escape was reversed by DNGR‐1 blockade with a neutralizing antibody, indicating that this effect is DNGR‐1 dependent. Data analyzed by two‐way ANOVA with Tukey's post hoc test (n = 3). (H) Fluorescence microscopy of dendritic cells expressing mCherry–lysenin, treated with or without fangchinoline (5 µM). Fangchinoline increased lysenin fluorescence surrounding phagosomes. Scale bars, 5 µm. Nuclei stained with Hoechst 33342. Analyzed by two‐tailed unpaired t‐test (n = 10, indicates 10 randomly selected microscopic fields). (I) Schematic of the cytochrome c–bead assay used to assess phagosomal membrane permeabilization. In this system, cytochrome c is conjugated to inert beads and phagocytosed by dendritic cells. If phagosomal membranes become permeable, cytochrome c is released into the cytosol, triggering apoptosis and serving as a functional readout of membrane disruption. (J) Fangchinoline (5 µM) enhanced phagosomal cytochrome c–induced apoptosis in MutuDCs, as measured by PI staining. Data analyzed by two‐way ANOVA with Tukey's post hoc test (n = 3). (K) Syk inhibition with R406 (1 µM) reversed fangchinoline‐induced ROS accumulation in MutuDC phagosomes. One‐way ANOVA (n = 3); ***p < 0.001. (L) Syk inhibition reversed fangchinoline‐enhanced cross‐presentation in OVA‐loaded MutuDCs, as assessed by H‐2K^b^–SIINFEKL staining. Data analyzed by two‐way ANOVA with Tukey's post hoc test (n = 3). (M) Nox2 inhibition with DPI (100 nM) similarly blocked fangchinoline‐induced enhancement of cross‐presentation. Data analyzed by two‐way ANOVA with Tukey's post hoc test (n = 3). (N) Scavenging phagosomal ROS with α‐tocopherol (25 µM) reduced fangchinoline‐induced phagosomal permeabilization, as shown by Cyto c–bead–induced apoptosis. Data analyzed by two‐way ANOVA with Tukey's post hoc test (n = 3). (O) α‐Tocopherol treatment also reversed fangchinoline‐induced enhancement of H‐2K^b^–SIINFEKL presentation in OVA‐loaded MutuDCs. Data analyzed by two‐way ANOVA with Tukey's post hoc test (n = 3). Fan, fangchinoline; G‐BMDC, GM‐CSF/IL‐4–derived bone marrow–derived dendritic cells; αDNGR‐1, anti‐DNGR‐1 antibody; PI, propidium iodide; MFI, mean fluorescence intensity; α‐toc, α‐tocopherol. *p < 0.05; ** *p* < 0.01; ***p < 0.001; ns: not significant.

To elucidate the downstream mechanism by which fangchinoline promotes dendritic cell (DC) antigen cross‐presentation, we investigated its effect on the DNGR‐1–Syk–Nox2 signaling axis (Figure 7A). Given the established role of spleen tyrosine kinase (Syk) in regulating DC function [12], we examined whether fangchinoline could activate this pathway. Immunoblotting revealed a concentration‐dependent increase in Syk phosphorylation at Tyr348 and Tyr352 following fangchinoline treatment, indicating robust activation of Syk (Figure 7B). This activation was significantly diminished by a DNGR‐1 neutralizing antibody, supporting its receptor dependence (Figure 7C).

We next assessed the impact of fangchinoline on phagosomal reactive oxygen species (ROS) levels, a downstream output of Syk signaling [42]. Flow cytometric analysis using OxyBURST‐labeled microspheres showed a time‐dependent increase in phagosomal ROS production in granulocyte‐macrophage colony‐stimulating factor–derived bone marrow dendritic cells (GM‐BMDCs) after fangchinoline treatment (Figure 7D).

To determine whether this was associated with enhanced export of endocytic cargo into the cytosol, we employed a saporin–puromycin assay [26] (Figure 7E). Fangchinoline significantly increased endosome‐to‐cytosol escape in MutuDCs (Figure 7F), and this effect was markedly reversed by antibody‐mediated blockade of DNGR‐1 (Figure 7G), indicating that fangchinoline promotes cytosolic access of internalized cargo in a DNGR‐1‐dependent manner. The flow‐cytometric gating strategy for this assay is shown in Figure S7A. Importantly, in the absence of saporin, neither fangchinoline nor DNGR‐1 blockade significantly altered puromycin labeling (Figure S7B), supporting the specificity of the assay. Consistent with increased phagosomal membrane destabilization, fangchinoline also enhanced Galectin‐3 accumulation, a marker of damaged phagosomal membranes [12, 43, 44] (Figure S7C). To further visualize phagosomal membrane disruption, we utilized a stable MutuDC line expressing mCherry–lysenin, a fluorescent reporter of membrane cholesterol exposure. Fangchinoline markedly increased lysenin accumulation surrounding phagosomes, consistent with enhanced membrane permeability (Figure 7H). Control experiments using cells expressing mCherry alone showed no such localization, confirming probe specificity (Figure S7D).

To functionally validate these findings, we employed a cytochrome c–bead assay to assess phagosomal membrane integrity [12, 45] (Figure 7I). In this system, MutuDCs phagocytose beads conjugated with cytochrome c, which, if released into the cytosol due to phagosomal leakage, triggers apoptosis. Fangchinoline treatment significantly increased bead‐induced apoptosis in MutuDCs, as measured by propidium iodide staining, indicating increased phagosomal permeability and rupture (Figure 7J).

Mechanistically, inhibition of Syk with R406 abolished the fangchinoline‐induced increase in phagosomal ROS (Figure 7K), suppressed Galectin‐3 recruitment to phagosomes (Figure S7E), and reversed the enhancement of antigen cross‐presentation, as measured by H‐2K^b^–SIINFEKL complex formation (Figure 7L). Similarly, blocking Nox2 using the NADPH oxidase inhibitor DPI attenuated fangchinoline‐induced cross‐presentation (Figure 7M), highlighting the role of ROS‐producing machinery. Finally, scavenging phagosomal ROS with α‐tocopherol suppressed both cytochrome c–induced apoptosis (Figure 7N) and antigen cross‐presentation (Figure 7O), further supporting the link between ROS‐dependent phagosomal permeabilization and enhanced cross‐presentation.

Collectively, these results demonstrate that fangchinoline enhances antigen cross‐presentation in dendritic cells by triggering a DNGR‐1–Syk–Nox2–ROS signaling cascade. This pathway promotes localized ROS accumulation within phagosomes, leading to membrane permeabilization and facilitating the translocation of antigens into the cytosol via the phagosome‐to‐cytosol (P2C) route. The resultant boost in cross‐presentation may contribute to improved T cell priming and anti‐tumor immunity within the melanoma microenvironment.

### 2.8 Fangchinoline Enhances PD‐1 Blockade Efficacy and Promotes Human Dendritic Cell–Mediated CD8^+^ T Cell Responses

**FIGURE 8:**
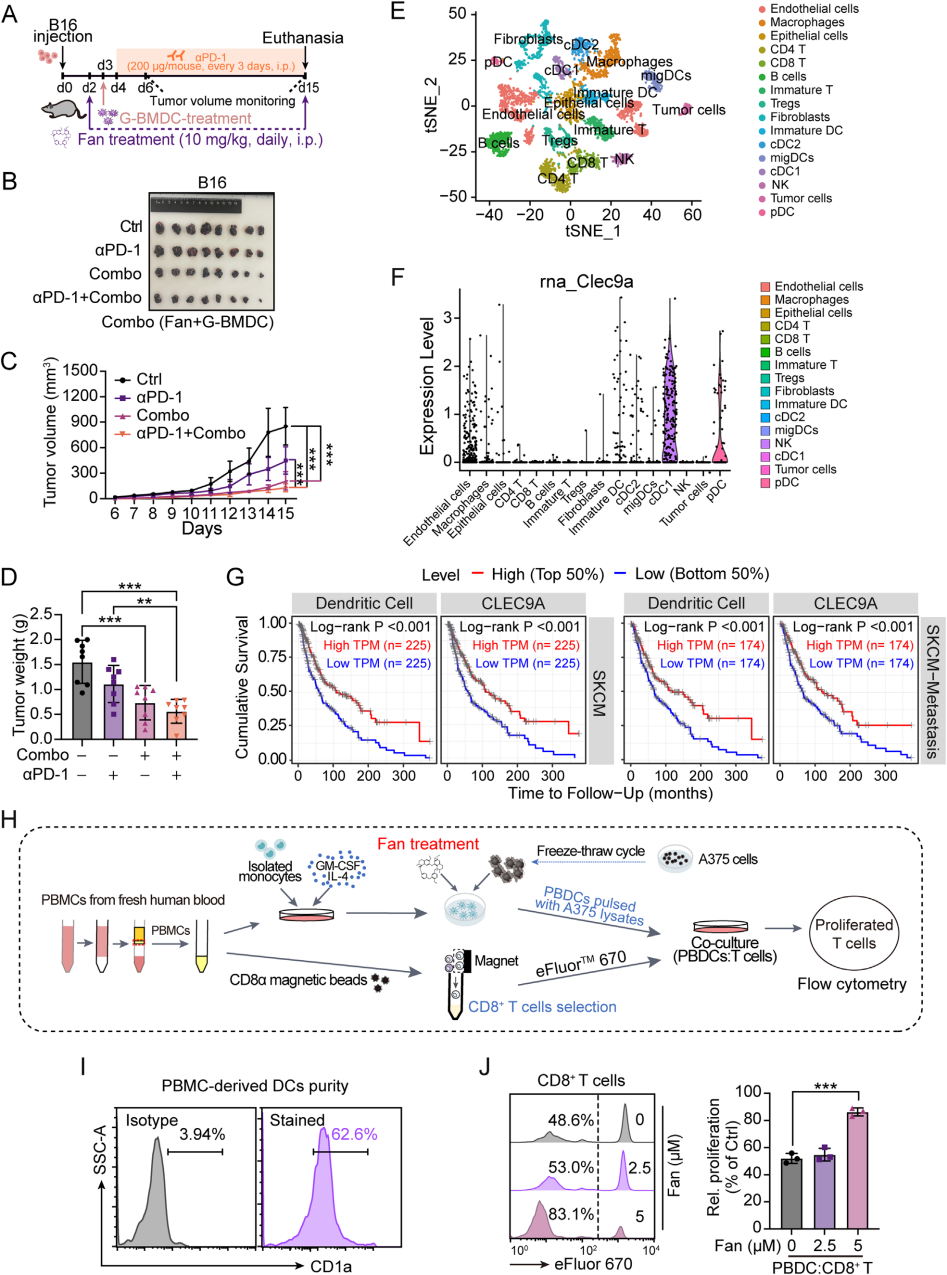
Fangchinoline potentiates PD‐1 blockade therapy and promotes human DC‐driven CD8^+^ T cell responses. (A) Schematic of the in vivo therapeutic regimen. C57BL/6 mice were subcutaneously inoculated with B16 melanoma cells (2 × 10^5^) on day 0. Fangchinoline (10 mg/kg) was administered daily via intraperitoneal injection starting on day 2. GM‐CSF/IL‐4–induced bone marrow–derived dendritic cells (G‐BMDCs) were injected into the right axilla on day 3. Anti–PD‐1 (αPD‐1; 200 µg/mouse) was given every 3 days starting from day 4. (B–D) Tumor growth was monitored from day 6. Tumor volumes (C) and final tumor weights (D) were significantly reduced in the combination treatment groups compared to monotherapies. Representative tumor images (B) and quantification shown. Data analyzed by two‐way ANOVA with Tukey's post hoc test (n = 8). (E,F) Single‐cell RNA‐seq analysis revealed that Clec9a expression is enriched in dendritic cells within the melanoma microenvironment. (G) TIMER database analysis showed that both dendritic cell abundance and CLEC9A expression positively correlate with overall survival in melanoma patients across multiple cohorts (log‐rank p < 0.001). (H) Schematic of the human PBMC‐derived DC (PBDC) co‐culture assay. Monocytes isolated from human PBMCs were differentiated into DCs and pulsed with soluble antigens from A375 melanoma cells in the presence or absence of fangchinoline (2.5 or 5 µM). PBDCs were then co‐cultured with eFluor 670–labeled CD8^+^ T cells to assess T‐cell proliferation. (I) Flow cytometry analysis confirmed the purity of human PBDCs based on CD11a and CD1a expression. (J) T‐cell proliferation was assessed by eFluor 670 dilution. Fangchinoline significantly enhanced CD8^+^ T cell proliferation in a dose‐dependent manner. Analyzed by one‐way ANOVA with Tukey's post hoc test (n = 3). Fan, fangchinoline; G‐BMDC, GM‐CSF/IL‐4–induced bone marrow–derived dendritic cell; αPD‐1, anti–PD‐1 antibody; PBDC, PBMC‐derived dendritic cell. *p < 0.05; ** *p* < 0.01; ***p < 0.001; ns: not significant.

To evaluate the therapeutic potential of fangchinoline in enhancing immune checkpoint blockade, we investigated its combinatorial effects with GM‐CSF/IL‐4–derived bone marrow dendritic cells (G‐BMDCs) and anti–PD‐1 antibody in a murine melanoma model. C57BL/6 mice bearing subcutaneous B16 melanoma tumors were treated with daily intraperitoneal injections of fangchinoline, local administration of G‐BMDCs, and intraperitoneal anti–PD‐1 every 3 days (Figure 8A). Compared with monotherapies, the combination of fangchinoline, G‐BMDCs, and anti–PD‐1 significantly suppressed tumor progression, as indicated by reduced tumor volumes and final tumor weights (Figure 8B–D).

To further investigate the underlying mechanism, we analyzed single‐cell RNA‐seq data from murine melanoma tissues. Clec9a, which encodes DNGR‐1, was predominantly expressed in dendritic cells within the melanoma microenvironment (Figure 8E,F), supporting the relevance of the DNGR‐1 pathway in DC‐driven antitumor immunity. In clinical datasets, analysis using the TIMER database revealed that both dendritic cell abundance and CLEC9A expression positively correlated with overall survival across multiple cohorts of patients with primary or metastatic melanoma (Figure 8G). These results suggest that DNGR‐1–expressing DCs are associated with favorable prognosis and highlight the clinical significance of this axis.

We further observed that fangchinoline not only enhances antitumor immunity via DNGR‐1 activation, but also induces a phenotypic shift in G‐BMDCs toward relatively higher DNGR‐1 expression, potentially enhancing their immunostimulatory capacity. These findings indicate that pharmacological targeting of DNGR‐1 in dendritic cells by fangchinoline may represent a promising immunotherapeutic approach.

To assess the translational relevance of these findings in human immune cells, we isolated monocytes and lymphocytes from human peripheral blood. Monocytes were differentiated into peripheral blood monocyte–derived dendritic cells (PBDCs) by cytokine induction, and CD8^+^ T cells were purified by magnetic sorting (Figure 8H). PBDC purity was confirmed by flow cytometry based on CD1a expression (Figure 8I). We then evaluated the effect of fangchinoline on human DC‐mediated T cell activation in a co‐culture system, in which PBDCs were pulsed with soluble melanoma antigens and incubated with eFluor 670–labeled CD8^+^ T cells. Fangchinoline treatment significantly enhanced CD8^+^ T cell proliferation in a dose‐dependent manner, as assessed by dye dilution (Figure 8J). In contrast, when moDCs without A375 tumor cell lysate loading were co‐cultured with autologous CD8^+^ T cells under the same conditions, minimal T cell proliferation was observed both before and after fangchinoline treatment. This further confirmed that fangchinoline‐induced T cell proliferation is antigen‐specific (Figure S8).

Together, these results support that fangchinoline enhances the efficacy of PD‐1 blockade in vivo and promotes human dendritic cell–mediated CD8^+^ T cell responses. By engaging DNGR‐1‐dependent antigen‐processing pathways and enhancing dendritic cell cross‐presentation, fangchinoline emerges as a promising immunomodulatory agent with translational potential in cancer immunotherapy.

## 3 Discussion

Dendritic cell (DC)–based vaccines represent a highly promising strategy in cancer immunotherapy, particularly for melanoma, due to their ability to initiate tumor‐specific CD8^+^ T cell responses [7, 8, 32]. However, their clinical efficacy remains limited by low response rates, the immunosuppressive tumor microenvironment, and, in some cases, immune‐related adverse events [29, 46]. As central orchestrators of innate and adaptive immunity, DCs are uniquely equipped to cross‐present exogenous antigens via MHC‐I to CD8^+^ cytotoxic T lymphocytes [47, 48, 49]. Enhancing this cross‐presentation capability is therefore a key objective in improving both DC vaccine efficacy and the induction of de novo T cell responses during immune checkpoint blockade therapy.

In this study, we identify fangchinoline, a natural bisbenzylisoquinoline alkaloid, as a previously unrecognized small‐molecule modulator of DC function. Fangchinoline enhances antigen cross‐presentation and anti‐tumor immunity by engaging DNGR‐1 (CLEC9A), a C‐type lectin receptor selectively expressed on conventional type 1 DCs (cDC1s). While DNGR‐1 has been previously implicated in sensing necrotic cells and facilitating uptake of cell‐associated antigens [12, 18, 50, 51], our study provides the first evidence that a small molecule can directly bind DNGR‐1 and functionally modulate its activity. Using surface plasmon resonance, molecular docking, and probe labeling, we identified a binding pocket composed of Trp209, Glu225, and Arg226, whose functional relevance was confirmed by site‐directed mutagenesis (Figure 6; Figure S6). These structural insights align with previous functional evidence showing that interference with DNGR‐1—via shRNA knockdown, antibody blockade, or Clec9a deficiency—abolished fangchinoline‐induced cross‐presentation (Figure 3C,D,K,L). While further studies are needed to establish its clinical utility, fangchinoline is, to our knowledge, the first reported small molecule to directly and functionally target DNGR‐1 in dendritic cells.

Mechanistically, our data support a model in which fangchinoline engages and enhances DNGR‐1‐associated Syk–Nox2 signaling, resulting in localized reactive oxygen species (ROS) generation within phagosomes (Figure 7). Nox2, a core component of the phagosomal NADPH oxidase complex, is well established as a major source of ROS during antigen processing, whereas Syk functions downstream of C‐type lectin receptor signaling to potentiate Nox2 activation and ROS production in dendritic cells [52, 53, 54]. Importantly, fangchinoline did not affect antigen uptake or phagocytosis (Figure S1E), indicating that its enhancement of cross‐presentation is unlikely to result from increased cargo internalization and instead reflects modulation of post‐uptake antigen‐processing steps. Consistent with this interpretation, fangchinoline increased phagosomal membrane permeability, as indicated by Galectin‐3 recruitment, lysenin probe redistribution, and cytochrome c–based assays (Figure 7, Figure S7C, and related panels), and promoted phagosome‐to‐cytosol escape (Figure 7F). This effect was reversed by DNGR‐1 blockade (Figure 7G), supporting the conclusion that enhanced cytosolic translocation of internalized material is DNGR‐1 dependent in the systems tested. These findings are consistent with previous studies showing that phagosomal ROS can destabilize membranes and facilitate antigen export into the cytosol, a critical step for cross‐presentation to CD8^+^ T cells [55, 56]. Moreover, the effects of fangchinoline on phagosomal membrane integrity and cytosolic escape were abolished by pharmacologic inhibition of Syk, Nox2 blockade, or ROS scavenging (Figure 7K–O, Figure S7E), further supporting involvement of the DNGR‐1–Syk–Nox2–ROS pathway. Notably, unlike broad immune activators, fangchinoline selectively enhanced phagosomal ROS and antigen‐processing capacity without inducing MutuDCs maturation or inflammatory cytokine production, as evidenced by unchanged expression of MHC‐II, CD80, CD86, and IL‐12/23p40 (Figure S1H–J). Together, these data identify fangchinoline as a selective enhancer of antigen cross‐presentation that acts primarily by promoting DNGR‐1‐dependent phagosomal destabilization and antigen escape into the cytosol, rather than by increasing antigen uptake or broadly activating conventional DC maturation pathways.

A particularly important finding of this study is the subset‐context‐dependent response of DCs to fangchinoline. GM‐CSF/IL‐4–induced BMDCs (G‐BMDCs), which exhibit low basal DNGR‐1 expression (Figure 5C) [40], responded robustly to fangchinoline, showing increased IRF8 expression and selected cDC1‐associated markers, including DNGR‐1 and XCR1 (Figure 5D–H) [15, 57]. Functionally, this was associated with enhanced ROS regulation (Figure 7D), increased cross‐presentation (Figure 1E), and improved T cell–mediated tumor control (Figure 2A–D,M–O). In contrast, Flt3L‐induced BMDCs (F‐BMDCs), which naturally express high levels of DNGR‐1 (Figure 5B), showed minimal therapeutic benefit and, after prolonged exposure, exhibited increased apoptosis (Figures 2E–H and 5L). This apoptotic response was largely reversed by DNGR‐1 blockade, implicating a DNGR‐1‐dependent stress response in its induction (Figure 5L). These findings underscore that the differentiation state and baseline receptor expression of DC subsets critically influence their responsiveness to pharmacological modulation, with G‐BMDCs representing a more plastic and tunable platform in this experimental setting.

These subset‐specific effects also support translational relevance. In melanoma patient datasets, reduced CLEC9A expression and lower DC abundance were significantly associated with poor prognosis (Figure 8G). In addition, fangchinoline enhanced CD8^+^ T cell activation in co‐cultures with human peripheral blood–derived DCs (Figure 8H–J), supporting functional activity in a human ex vivo setting. Given the differential responsiveness of DC subsets across DNGR‐1 expression states, this receptor warrants further evaluation as a biomarker to help guide patient stratification and the design of DC‐focused therapeutic approaches.

The therapeutic potential of fangchinoline was further supported in vivo. Although fangchinoline or G‐BMDCs alone had limited efficacy, their combination significantly suppressed tumor growth and increased CD8^+^ T cell infiltration and effector function (Figure 2A–D,M–O). These effects were associated with increased expression of selected cDC1‐associated markers and a cross‐presentation‐associated program in G‐BMDCs (Figure 5D–K), together with enhanced DNGR‐1‐dependent antigen cross‐presentation and CD8^+^ T cell priming (Figure 3C–D,F,H–J). When combined with anti–PD‐1 therapy, fangchinoline further improved tumor control (Figure 8A–D), consistent with strengthened DC–T cell interactions within the tumor microenvironment. Notably, the fangchinoline–G‐BMDC combination alone achieved tumor suppression comparable to anti–PD‐1 monotherapy in this model (Figure 8B–D), supporting further investigation of this DC‐focused immunotherapeutic strategy.

These preclinical findings support further translational exploration of fangchinoline. By enhancing DC cross‐presentation without requiring exogenous antigen loading, fangchinoline may offer a complementary strategy to strengthen current DC vaccine platforms, which often depend on antigen identification, isolation, and in vitro pulsing [39, 58]. By promoting presentation of tumor‐derived antigens in situ, fangchinoline may also help address immune escape associated with tumor heterogeneity. In this context, DNGR‐1 expression warrants further evaluation as a functional biomarker for selecting potent cross‐presenting DCs ex vivo or for stratifying responses according to tumor‐infiltrating DC profiles.

In addition to these insights, our data also address potential safety concerns. Fangchinoline did not induce MutuDCs maturation or pro‐inflammatory cytokine release under steady‐state conditions (Figure S1H–J), suggesting a favorable immunological safety profile in the assays tested. Moreover, its effects on CD8^+^ T cell activation were observed without overt systemic toxicity in the murine models examined. Several limitations should be noted. First, the in vivo treatment schedule used in this study represents an early therapeutic intervention rather than a fully established palpable‐tumor treatment model. Fangchinoline treatment and DC transfer were initiated shortly after tumor inoculation in the rapidly progressing B16 melanoma model, which is useful for evaluating early immune modulation and therapeutic synergy, but does not establish definitive efficacy against advanced or clearly palpable tumors. Future studies using delayed treatment initiation in mice bearing measurable tumors will be important to further evaluate the translational therapeutic potential of this strategy. Second, although our data support direct and functional involvement of DNGR‐1, they do not establish a definitive receptor‐active structural mechanism at atomic resolution. Third, although the dead‐cell antigen model better reflects canonical DNGR‐1 biology, the soluble OVA assays should be interpreted primarily as tractable readouts of shared post‐uptake antigen‐processing steps rather than as direct evidence of DNGR‐1‐mediated cargo capture; importantly, fangchinoline showed a stronger DNGR‐1‐dependent effect in the dead‐cell context. Relatedly, an inherent limitation of B16‐OVA–based dead‐cell antigen systems is that B16‐OVA cells may generate endogenous H‐2K^b^–SIINFEKL complexes. Thus, part of the H‐2K^b^–SIINFEKL signal detected on DCs after co‐culture could theoretically reflect acquisition of preformed peptide–MHC‐I complexes from tumor cells, a process known as cross‐dressing, rather than exclusively de novo antigen processing and cross‐presentation by DCs. Nevertheless, the DNGR‐1 dependence, lack of effect on antigen uptake, modulation of phagosomal ROS and membrane permeability, and functional OT‐I T cell activation data support the interpretation that enhanced de novo cross‐presentation through post‐uptake processing mechanisms contributes substantially to the observed response. Future studies using H‐2K^b^‐, β2‐microglobulin‐, or TAP‐deficient OVA‐expressing tumor cells, or other systems that minimize or eliminate preformed peptide–MHC‐I transfer, will help more precisely distinguish de novo cross‐presentation from cross‐dressing. Fourth, while fangchinoline increased selected cDC1‐associated markers in GM‐CSF/IL‐4‐derived cultures, these data should not be interpreted as proof of lineage conversion. Fifth, although quality‐control analyses of transferred F‐BMDCs did not reveal a systematic compositional bias sufficient to explain the therapeutic outcomes, the absolute number of XCR1^+^ cells was not prospectively normalized across all adoptive‐transfer experiments. Finally, we did not perform unbiased RNA‐seq or module‐level transcriptomic analyses. Instead, the PCR array was used as a targeted gene‐expression assay to examine selected DC activation and cross‐presentation‐associated genes. Therefore, these data should be interpreted as targeted supporting evidence rather than transcriptome‐wide evidence. Future studies using purified DC subsets, direct antigen‐trafficking imaging, long‐term efficacy models in established tumors, immunological memory assays, unbiased transcriptomic profiling, and tissue‐specific biodistribution analyses will further refine the mechanistic and translational implications of fangchinoline.

In summary, this study identifies fangchinoline as a novel small‐molecule modulator of DNGR‐1 that enhances antigen cross‐presentation, induces selected cross‐presentation‐associated features in dendritic cell systems, and potentiates anti‐tumor immunity. Mechanistically, our data support involvement of the DNGR‐1–Syk–Nox2–ROS axis in promoting phagosomal membrane permeabilization, enabling antigen translocation into the cytosol—a critical step for cross‐presentation via the cytosolic pathway—and facilitating CD8^+^ T cell priming. By promoting DC cross‐presentation and CD8^+^ T cell activation, including in human ex vivo co‐culture systems, fangchinoline warrants further evaluation as a DNGR‐1‐targeting immunomodulatory candidate for melanoma, particularly in combination with checkpoint blockade or DC‐based strategies.

## 4 Materials and Methods

### 4.1 Cell Culture and Materials

Mouse melanoma cell lines (B16 and B16‐OVA) and immortalized mouse dendritic cells (MutuDC, MutuDC1940) were obtained from Cresbo Biotechnology and Zhejiang Meisen CTCC, respectively. The human melanoma cell line A375 was purchased from Procell (CL‐0014). B16, B16‐OVA, and MutuDC cells were cultured in RPMI 1640 medium (11875119, Gibco) supplemented with 10% fetal bovine serum (FBS; ExCell, FSP500), 1% penicillin–streptomycin (P1400, Solarbio), and 50 µM β‐mercaptoethanol (only for MutuDC). A375 and HEK293T cells were maintained in DMEM (11965092, Gibco) supplemented with 10% FBS and 1% penicillin–streptomycin. All cells were cultured at 37°C in a humidified incubator containing 5% CO_2_. Fangchinoline (T3122, purity 99.98%) was purchased from TargetMol and prepared in DMSO for in vitro studies or dissolved in vehicle for in vivo administration.

### 4.2 Melanoma Transplantation Experiments and In Vivo Antibody Injection

All animal experiments were approved by the Animal Ethics Committee of Guangzhou University of Chinese Medicine (Approval No. 20230514001). Male C57BL/6 mice (7–8 weeks old, 18–21 g) were obtained from the Guangdong Medical Laboratory Animal Center and housed under specific pathogen‐free (SPF) conditions with access to sterile food and water ad libitum. After 1 week of acclimatization, mice were subcutaneously inoculated in the right axilla with 2 × 10^5^ B16 cells or 4 × 10^5^ B16‐OVA cells. In some experiments, 5 × 10^4^ bone marrow‐derived dendritic cells (BMDCs) were co‐injected into the tumor‐draining axilla. Fangchinoline was administered intraperitoneally at a dosage of 10 mg/kg once daily. On day 4 post‐inoculation, therapeutic antibodies including anti‐CD8α (BE0061, BioXcell), anti‐DNGR‐1 (BE0305, BioXcell), and anti–PD‐1 (BE0146, BioXcell) were administered intraperitoneally at 200 µg per mouse. Tumor volumes were measured daily starting from day 5 or 6. All measurements were performed in a blinded manner, with the individual responsible for assessing tumor size unaware of the treatment group allocations, in order to minimize potential bias.

### 4.3 Generation of Dendritic Cell–Specific DNGR‐1 Ablation Mouse Model

CD11c‐tdTomato‐DTR transgenic mice (Itgax‐2A‐tdTomato‐2A‐DTR, NM‐KI‐204992) and Clec9a‐knockout (Clec9a‐KO, NM‐KO‐190204) mice on a C57BL/6 background were purchased from Shanghai Model Organisms Center, Inc. Genotyping was performed using primers listed in Table S1. Mice used in this study were 7–8 weeks old, weighed 18–21 g, and were sex‐matched littermates. Animals were housed under SPF conditions with ad libitum access to sterile food and water. For tumor inoculation, mice were subcutaneously injected in the right axilla with 2 × 10^5^ B16 cells on day 0. To deplete endogenous CD11c^+^ cells, CD11c‐tdTomato‐DTR mice received intraperitoneal injections of diphtheria toxin (DT; D0564, Sigma)30 ng/g on days −1, 0, and every 3 days thereafter. On day 3, 5 × 10^4^ BMDCs from either wild‐type or Clec9a‐KO donors were injected into the tumor‐draining axilla. All animal procedures were approved by the Animal Ethics Committee of Guangzhou University of Chinese Medicine (Approval No. 20230514001) and conducted in accordance with institutional guidelines for animal care.

### 4.4 Preparation of Bone Marrow‐Derived Dendritic Cells and CD8^+^ T Cells

GM‐CSF/IL‐4–Derived BMDCs: Bone marrow cells were flushed from the femurs and tibias of C57BL/6 mice and cultured in RPMI 1640 medium supplemented with 10% FBS (ExCell, FSP500), 20 ng/mL GM‐CSF (CK02, Novaprotein), 10 ng/mL IL‐4 (CK74, Novaprotein), and 1% penicillin–streptomycin. Fresh cytokine‐containing medium was added every 2 days. On day 6, BMDC maturation was induced by supplementing the culture with 500 ng/mL lipopolysaccharide (LPS; L4516, Sigma‐Aldrich) for 48 h before harvest.

Flt3L‐Induced BMDCs: Bone marrow cells were seeded at 2 × 10^7^ cells/mL in 6‐well plates with 4 mL RPMI 1640 medium containing 10% FBS, 80 ng/mL Flt3 ligand (Flt3L; 250‐31L, PeproTech), 50 µM β‐mercaptoethanol, and 1% penicillin–streptomycin. On days 5 and 8, half of the culture medium was replaced with fresh medium containing Flt3L and β‐mercaptoethanol. Cells were harvested between days 7 and 13 for downstream experiments.

CD8^+^ T Cell Isolation from OT‐I Mice: OT‐I transgenic mice, which express a T‐cell receptor specific for OVA (257–264) presented by H‐2K^b^, were obtained from GENEANDPEASE. Spleens were harvested and mechanically dissociated, and lymphocytes were isolated using a lymphocyte separation solution (7211011, Dakewe Biotech). CD8^+^ T cells were enriched using CD8α (Ly‐2) MicroBeads (130‐117‐044, MACS) and MACS buffer (130‐091‐222‐1, Miltenyi Biotec) followed by magnetic column separation. Purified CD8^+^ T cells were cultured in complete medium supplemented with IL‐2 (212‐12‐100, PeproTech) for activation and subsequent use.

### 4.5 Antigen Cross‐Presentation Assay

BMDCs and MutuDCs were cultured in 6‐well plates and incubated for 24 h to allow for cell adherence and stabilization. Subsequently, the cells were pulsed with ovalbumin (OVA) at a concentration of 200 µg/mL, followed by treatment with varying concentrations of fangchinoline for 24 h to assess dose‐dependent effects on antigen presentation. Post‐treatment, cells were washed with phosphate‐buffered saline (PBS), detached using trypsin, and stained with an H‐2K^b^–SIINFEKL antibody (PE, 17574382, Invitrogen) to detect OVA‐derived peptide presentation on MHC class I molecules.

For MutuDCs, unstained controls were used to define background fluorescence and guide positive gate placement in the single‐stain assay, in a manner analogous to fluorescence‐minus‐one (FMO) controls, particularly for H‐2K^b^–SIINFEKL staining. The mean fluorescence intensity (MFI) of H‐2K^b^–SIINFEKL staining was measured, and background fluorescence estimated from the corresponding unstained, isotype, or background control was subtracted before normalization to the control group. In BMDC experiments, cells were co‐stained with a CD11c antibody (FITC, 117306, BioLegend) to identify BMDCs, together with the corresponding H‐2K^b^–SIINFEKL staining controls. Following background subtraction, normalized MFI values were used to assess antigen cross‐presentation.

### 4.6 Dead Cell Antigen Cross‐Presentation Assay

B16‐OVA and B16 cells were subjected to repeated freeze–thaw cycles to induce cell death, consisting of rapid freezing in liquid nitrogen for 5 min followed by thawing in a 37°C water bath for 5 min, repeated five times. To preserve the structure of cellular debris, the suspensions were collected by low‐speed centrifugation (1000 rpm, 5 min), and the resulting pellets containing dead cells and cell fragments were used as tumor antigens. Dead tumor cells were then co‐incubated with MutuDCs at a ratio of 3:1. After incubation, the cross‐presentation of OVA‐derived peptide on MHC class I was assessed by flow cytometry using H‐2K^b^–SIINFEKL staining, and antigen presentation was quantified as the mean fluorescence intensity (MFI).

### 4.7 Measurement of T‐Cell Proliferation and IFN‐γ Release

T Cell Proliferation: Proliferation of OT‐I CD8^+^ T cells or human peripheral blood T cells was assessed using the eFluor 670 proliferation dye (65‐0840‐90, Invitrogen). Following isolation, cells were washed twice with PBS to remove residual serum and resuspended at 2× the target concentration. A 10 µM eFluor 670 dye solution was added at 2 µL/mL, and cells were incubated at 37°C for 10 min in the dark. The reaction was quenched by adding 4–5 volumes of cold complete medium (containing ≥10% FBS), followed by a 5‐min incubation on ice. Cells were then centrifuged and washed three times with complete medium before use. Proliferation was analyzed by flow cytometry based on dye dilution.

IFN‐γ Release: MutuDCs were treated with 200 µg/mL OVA and fangchinoline (2.5 or 5 µM) for 10 h, with or without DNGR‐1 blockade. Treated DCs were then co‐cultured with OT‐I CD8^+^ T cells at appropriate ratios for 12 h. Supernatants were collected after centrifugation and analyzed for interferon‐gamma (IFN‐γ) levels using an ELISA kit (RK00019, Abclonal), following the manufacturer's instructions.

### 4.8 Analysis of B16‐OVA Cell Apoptosis​

To assess tumor cell apoptosis, B16‐OVA cells were co‐cultured with OT‐I CD8^+^ T cells. Apoptotic cells were labeled using propidium iodide (PI) apoptosis staining solution. Since MutuDCs intrinsically express GFP, and CD8^+^ T cells were stained with anti‐CD8α antibody (APC, 100712, BioLegend), these populations were excluded by gating out GFP^+^ and CD8α^+^ cells. The remaining B16‐OVA cell population was analyzed by flow cytometry to quantify the proportion of PI^+^ apoptotic cells.

### 4.9 Isolation of Draining Lymph Node and Tumor‐Infiltrating Lymphocytes​

Draining lymph nodes and tumor tissues were harvested and enzymatically digested in DMEM supplemented with 5% FBS (Seradigm, 3100), 2 mg/mL collagenase D (11088866001L, Sigma), 1 mg/mL collagenase IV (C5138‐1G, Sigma), and 40 µg/mL DNase I (10104159001, Sigma). Samples were incubated at 37°C with gentle shaking for 30–60 min. The resulting suspensions were centrifuged at 400 × g for 5 min, and pellets were resuspended in 70% Percoll (17089109‐1, GE Healthcare). A 40% Percoll overlay was added, and the mixture was centrifuged at 500 × g for 20 min at room temperature with minimal acceleration and deceleration. Mononuclear cells at the interface were collected and filtered through a 70 µm strainer to remove debris.

For flow cytometric analysis, isolated lymphocytes were first stained with the viability dye FVS620 (564996, BD Biosciences) at a 1:1000 dilution for 10 min at room temperature. Cells were then blocked with anti‐mouse CD16/32 antibody (553141, BD Biosciences) for 5 min at 4°C to prevent non‐specific binding. Subsequently, cells were stained with the following antibodies (1:100 dilution): CD45 (APC‐Cy7, 553082, BD Biosciences), CD3e (PE‐Cy5.5, 551163, BD Biosciences), CD8α (APC, 100712, BioLegend), CD11c (FITC, 117306, BioLegend), XCR1 (APC, 148206, BioLegend), and H‐2K^b^–SIINFEKL (PE, 17574382, Invitrogen). After 20 min of incubation at room temperature in the dark, cells were washed and analyzed using a BD LSR Fortessa flow cytometer. Data were analyzed to identify and quantify tumor‐infiltrating lymphocyte subsets based on surface marker expression.

### 4.10 PCR Array Plate

Gene expression profiling was conducted using the Dendritic and Antigen‐Presenting Cell PCR Array (WC‐MRNA0040‐M, WcGene Biotechnology). Total RNA was extracted and reverse transcribed into cDNA. The cDNA was diluted to a final volume of 100 µL and mixed with qPCR master mix according to the manufacturer's protocol. The mixture was dispensed into the PCR array plate, centrifuged briefly at 2000 rpm for 20 s, and loaded into a real‐time PCR system. Gene expression levels were analyzed based on amplification curves to identify differentially expressed genes.

### 4.11 Analysis of Phagosomal ROS in BMDCs

BMDCs were seeded at a density of 1 × 10^6^ cells per well in 6‐well plates and treated under the following conditions: vehicle control, isotype control, fangchinoline (2.5 or 5 µM), and Syk inhibitor R406 (with or without fangchinoline). Negative and isotype control groups were not incubated with ROS‐sensitive dyes. For ROS detection, OxyBURST Green H2DCFDA‐SE (D2935, ThermoFisher)–labeled Polybead Amino Microspheres (3.00 µm, 17145‐5, Polysciences) were prepared the day before. Microspheres and BMDCs were co‐cultured at a 5:1 bead‐to‐cell ratio, and microspheres were incubated in a nitrogen atmosphere overnight. After 6 h of drug treatment, cells were washed and re‐plated in fresh medium containing dye‐labeled microspheres. Following a 1‐h incubation at 37°C, cells were collected, washed twice with flow cytometry buffer, and stained with CD11c antibody (PE, 557401, BD Biosciences) and isotype control (553954, BD Biosciences) for 20 min in the dark. Phagosomal ROS levels were quantified by measuring FITC fluorescence via flow cytometry. Data were analyzed to assess the effects of fangchinoline and R406 on ROS production.

### 4.12 Phagocytosis Assay

MutuDCs were seeded on glass coverslips in 12‐well plates at a density of 2.5 × 10^5^ cells per well and treated with fangchinoline (5 µM) or CpG (1 µM) as indicated. Amino‐modified polystyrene beads (3.00 µm, 17145‐5, Polysciences) were activated with glutaraldehyde, coated overnight with rabbit IgG (1 mg/mL, A7058, Beyotime), blocked with ethanolamine and BSA, and resuspended in complete medium. IgG‐opsonized beads were then added to cells at a bead‐to‐cell ratio of approximately 20:1 and incubated at 37°C for 2 h.

Cells were fixed with 4% paraformaldehyde and subjected to an inside‐out staining protocol to distinguish extracellular and internalized beads. Extracellular beads were labeled with Alexa Fluor 594‐conjugated anti‐rabbit IgG (ab150084, Abcam) before permeabilization, and total bead‐associated signals were labeled with Alexa Fluor 405‐conjugated anti‐rabbit IgG (ab175652, Abcam) after permeabilization. Coverslips were mounted and imaged by confocal microscopy. Internalized beads were defined as Alexa Fluor 405 single‐positive signals, whereas extracellular beads were defined as Alexa Fluor 594/Alexa Fluor 405 double‐positive signals. Phagocytosis was quantified by calculating the phagocytic index and phagocytic efficiency.

### 4.13 Saporin–Puromycin Escape Assay

MutuDCs were subjected to different pretreatments prior to the assay. Cells in the Fan and aDNGR‐1 groups were pretreated for 12 h; the cycloheximide group was treated with 10 µg/mL cycloheximide (S7418, Selleck) for 2 h; and the saporin group was treated with 0.1 mg/mL saporin (S9896, Merck) for 2 h. Subsequently, puromycin (T2219, TargetMol) was added to all groups at a final concentration of 0.01 mg/mL, and the cells were incubated for an additional 30 min. After treatment, the cells were harvested, fixed, permeabilized, and stained with an anti‐puromycin antibody (Alexa Fluor 647, 381508, BioLegend). Stained cells were then analyzed by flow cytometry.

### 4.14 Cytochrome c Release Assay

BMDCs were treated with 5 µM fangchinoline for 24 h. In parallel, Polybead Microspheres (3.00 µm, 17145, Polysciences) were coated with cytochrome c by incubating the beads overnight at 4°C in a 10 mg/mL cytochrome c solution. The microsphere‐to‐cell ratio was maintained at 5:1, and the bead concentration was 1.68 × 10^9^/mL. After coating, microspheres were washed twice with PBS and resuspended in complete culture medium.

Cytochrome c–coated microspheres were added to BMDC cultures in 12‐well plates that had been pretreated with fangchinoline and α‐tocopherol. After 24 h of incubation, cells were harvested and stained with PI apoptosis markers. Following centrifugation at 2000 rpm for 5 min, the extent of cytochrome c–induced apoptosis was analyzed by flow cytometry using standard protocols.

### 4.15 Biotin Labeling Assay for Detecting Fangchinoline Binding to DNGR‐1

To assess fangchinoline binding to DNGR‐1, the previously synthesized Fan‐probe was employed. MutuDCs were cultured in 10 cm dishes and divided into three groups: treated with control probe (Ctrl‐probe), Fan‐probe, or Fan‐probe combined with excess fangchinoline to evaluate competitive binding. All groups were incubated with their respective treatments for 20 min.

After treatment, cells were washed twice with PBS and exposed to UV light for 20 min to facilitate photo‐crosslinking. Cells were then lysed using RIPA buffer containing protease inhibitors, incubated on ice for 10 min, and centrifuged at 14 000 rpm for 10 min at 4°C. Supernatants were collected, and protein concentrations were determined using an enhanced BCA protein assay kit.

For click chemistry, 200 µg of protein from each sample was reacted with Biotin‐PEG3‐Azide (400 µM), TBTA (100 µM), TCEP (1 mM), and CuSO_4_·5H_2_O (1 mM) at room temperature for 3 h with continuous rotation. Biotinylated proteins were precipitated with acetone, resuspended in PBS, and incubated with 50 µL of Dynabeads M‐280 Streptavidin (11205D, Invitrogen) for 1 h at room temperature. After extensive washing, bound proteins were eluted by heating with 1× loading buffer at 100°C for 10 min and analyzed by Western blot to confirm specific binding of fangchinoline to DNGR‐1.

### 4.16 Surface Plasmon Resonance Analysis

Surface plasmon resonance was used to quantify the binding interaction between fangchinoline and recombinant mouse CLEC9A protein with a human Fc tag (SinoBiological, 51145‐M01H). CLEC9A protein was diluted in sodium acetate buffer (pH 4.0) and immobilized onto an S series CM5 sensor chip (Cytiva, 29149603) to a level of approximately 10 000 response units (RU). The reference flow cell (Fc3) was activated and blocked to ensure specificity. Fangchinoline was diluted to 3 µg/mL in 1× PBST and serially diluted twofold across at least five concentrations. Each sample was injected for 60 s (association phase), followed by 60 s of dissociation. Binding kinetics were analyzed using either a 1:1 Langmuir binding model or steady‐state affinity analysis, providing detailed characterization of fangchinoline–CLEC9A interaction dynamics.

### 4.17 Cellular Thermal Shift Assay

CETSA was performed to validate the intracellular interaction between fangchinoline and DNGR‐1. Membrane proteins were extracted from BMDCs using a commercial membrane protein extraction kit (Bestbio, BB‐3103). Cells were treated with 5 µM fangchinoline or left untreated for 20 min. After treatment, protein lysates were divided into nine equal aliquots and subjected to thermal denaturation at 67°C for 0, 30, 60, 90, 120, 150, 180, 210, and 240 s. Samples were then immediately cooled on ice. Each aliquot was then mixed with 5× SDS loading buffer and analyzed by Western blot to assess thermal stabilization of DNGR‐1. Enhanced thermal resistance in the fangchinoline‐treated group indicated direct ligand binding and structural stabilization of the receptor.

### 4.18 Rhodamine Fluorescence Labeling of Fangchinoline‐Binding Proteins

To visualize fangchinoline‐binding proteins, MutuDCs or Clec9a knockdown (shClec9a) MutuDCs were cultured in confocal dishes or 6‐well plates. Cells were then incubated with either the Ctrl‐probe or Fan‐probe for 10 min. Following probe treatment, cells were washed with PBS and irradiated with UV light for 10 min to activate the photo‐click reaction. Cells were then fixed with 4% paraformaldehyde for 20 min, washed, and incubated with a click chemistry reaction mixture containing RB‐PEG3‐N_3_ (400 µM), TBTA (100 µM), TCEP (1 mM), and CuSO_4_·5H_2_O (1 mM) for 15 min at room temperature. Nuclei were stained with Hoechst 33342 for 15 min. Cells were then imaged using a confocal laser microscope to visualize rhodamine fluorescence. For quantification, cells were trypsinized and analyzed by flow cytometry using the PE channel to measure Fan‐probe binding.

### 4.19 Detection of Lysenin Fluorescence Intensity

Two stable DC lines were generated—one expressing mCherry and the other co‐expressing mCherry and lysenin. According to a previously published method [12], cells were cultured in confocal dishes and co‐treated with 5 µM fangchinoline and Polybead Microspheres (at a 5:1 ratio of beads to DCs) for 24 h. After treatment, cells were washed with PBS, fixed with 4% paraformaldehyde for 20 min, and washed again. Nuclei were stained with Hoechst 33342 for 15 min. After a final PBS wash, Hank's Balanced Salt Solution (HBSS) was added. Cells were then imaged using a Zeiss fluorescence microscope, and lysenin^+^ phagocytic cells were counted. The data were presented as the ratio of lysenin^+^ cells to total bead^+^ cells (index).

### 4.20 Single‐cell RNA‐seq Data Processing and Analysis

Single‐cell RNA sequencing data from murine melanoma tumors were obtained from the ArrayExpress database (accession: E‐MTAB‐7427) [59] to investigate immune cell heterogeneity within the tumor microenvironment. Raw count matrices were aligned to the GRCm38/mm10 reference genome and processed using Seurat (v4.0) in R [60]. Quality control steps included filtering out cells with fewer than 300 detected genes, fewer than 1000 unique molecular identifiers (UMIs), or more than 12.5% mitochondrial gene content. Cells exhibiting abnormally high ribosomal or erythroid gene expression were also excluded. Potential doublets were identified and removed using DoubletFinder [61]. Gene expression data were log‐normalized, and highly variable genes were selected for downstream analysis. Dimensionality reduction was performed using principal component analysis (PCA), followed by graph‐based clustering and visualization with t‐distributed stochastic neighbor embedding (t‐SNE). Cell type annotation was based on the expression of canonical marker genes. Differential gene expression analysis between clusters was performed using the Wilcoxon rank‐sum test implemented in Seurat.

### 4.21 Evaluation of Human CD8^+^ T Cell Responses

A375 melanoma cells in logarithmic growth were harvested, washed, and resuspended at 10^7^–10^8^ cells/mL. Tumor soluble antigens (TSA) were prepared by four freeze–thaw cycles (−80°C for 2 h, followed by 37°C for 30 min), and the supernatant was collected by centrifugation, filtered through a 0.22 µm membrane, and stored at −80°C.

Peripheral blood mononuclear cells (PBMCs) were obtained from healthy adult male donors recruited from Zhongshan Hospital of Traditional Chinese Medicine Affiliated to Guangzhou University of Chinese Medicine (Zhongshan, China). Inclusion criteria for the healthy control group were as follows: (1) healthy adult males; (2) no use of antibiotics or other microbial agents within 2 weeks prior to blood collection; and (3) good compliance and provision of written informed consent. The clinical trial was approved by the Chinese Clinical Trial Registry (Approval No. ChiCTR2400082028) and conducted in accordance with ethical guidelines.

After Ficoll density gradient centrifugation, PBMCs were isolated and cultured in RPMI 1640 supplemented with GM‐CSF and IL‐4 to generate immature dendritic cells (PBDCs), which were matured with LPS stimulation. CD8^+^ T cells were isolated using magnetic‐activated cell sorting and cultured in IL‐2–containing medium. Mature PBDCs were incubated with TSA and fangchinoline, then co‐cultured with eFluor 670‐labeled CD8^+^ T cells. T cell proliferation was assessed by flow cytometry based on dye dilution.

### 4.22 Statistical Analysis

All data are presented as mean ± standard deviation (SD). For in vitro experiments, n represents independent biological replicates, defined as independent cell cultures prepared and treated on separate days. For in vivo experiments, n represents individual mice. No data points represent technical replicates unless explicitly stated in the figure legend. Comparisons between two groups were performed using unpaired two‐tailed Student's *t*‐tests. For comparisons involving three or more groups, one‐way ANOVA followed by Tukey's multiple comparisons test was used. For experiments involving two independent variables, two‐way ANOVA followed by Tukey's multiple comparisons test was applied. Statistical analyses were conducted using GraphPad Prism software. A *p* value < 0.05 was considered statistically significant.

## Author Contributions

Conceptualization, K.W., JY.X., F.L. and YF.S.; methodology, Y.L., ZY.R., J.Z. and CZ.L.; software, ZY.R., H.L., YJ.J. and JY.G.; validation, YL.L., D.C., MY.Z. and AQ.L.; formal analysis, Y.Z., YX.J. and BY.D.; investigation, Y.L., ZY.R., J.Z. and H.L.; resources, Y.W., DY.W., JY.G. and BY.D.; data curation, D.C., YX.J., MY.Z. and AQ.L.; writing – original draft preparation, K.W. and Y.L.; writing – review and editing, YF.S., F.L. and JY.X.; visualization, Y.L., ZY.R., J.Z.; supervision, YF.S. and F.L.; project administration, K.W.; funding acquisition, K.W. and JY.X.

## Funding

This work was supported by grants from the National Natural Science Foundation of China (Grant No. 82574775, 82104571), the Natural Science Foundation of Guangdong Province (Grant No. 2025A1515012216, 2025A1515012841), the Science and Technology Plan Project of Guangzhou of China (Grant No. 2023A04J2477, 2024A04J10028).

## Ethics Statement

The clinical trial was duly registered and received approval from the Chinese Clinical Trial Registry (Approval No. ChiCTR2400082028). All procedures involving animals were conducted in strict accordance with ethical standards and were formally approved by the Animal Ethics Committee of Guangzhou University of Chinese Medicine (Approval No. 20230514001).

## Consent

All participants involved in the study provided written informed consent for the publication of any associated data.

## Conflicts of Interest

The authors declare no conflicts of interest.

## Supporting information




**Supporting File**: advs76043‐sup‐0001‐SuppMat.pdf.

## Data Availability

The data supporting the findings of this study are available in the Supplementary Materials of this article. Additional de‐identified data are available from the corresponding author upon reasonable request, subject to applicable ethical and privacy restrictions. The single‐cell sequencing data used in this study are publicly available from the European Bioinformatics Institute database under accession number E‐MTAB‐7427^60^. No custom code was generated or used in this study.
