# Supplementary material for: Targeting DNGR‐1 with Fangchinoline Elevates Dendritic Cell Antigen Cross‐Presentation‐Mediated Antitumor Immunity in Melanoma
Source: Adv Sci (Weinh). 2026 Jun 11;13(50):e76043. doi: 10.1002/advs.76043 (PMC13336900; doi:10.1002/advs.76043)
Supplement: Supplementary file 1 [file ADVS-13-e76043-s001.pdf]

Supplementary Materials for

Targeting DNGR-1 with Fangchinoline Elevates Dendritic Cell Antigen Cross-Presentation-mediated Antitumor Immunity in Melanoma

Yuan Liao, Zhenyang Ren, Jing Zhou, He Luo, Yuling Li, Dan Chen, Yuxiao Jiang, Canzhe Li, Mengyi Zheng, Anqi Li, Yao Wang, Yang Zhang, Dingye Wang, Yanjun Jiang, Jiangyong Gu, Biaoyan Du, Yafei Shi, Fang Liu, Jianyong Xiao, Kun Wang

Corresponding author: wangkun@gzucm.edu.cn (K.W.); jianyongxiao@gzucm.edu.cn (JY.X.); fangliu@gzucm.edu.cn (F.L.); shiyafei@gzucm.edu.cn (YF.S.).

The PDF file includes:

Materials and Methods

Figs. S1 to S8

Tables S1 to S5

Materials and Methods

Flow Cytometry Analysis

Following co-culture or specific treatments, cells were harvested by trypsinization and washed twice with BD Stain Buffer to eliminate residual culture medium. Cell pellets were stained with appropriate fluorophore-conjugated antibodies at room temperature for 20 minutes in the dark to prevent photobleaching. Stained cells were analyzed using a BD LSR Fortessa™ flow cytometer. Data were processed with FlowJo software to quantify the expression levels and proportions of cells expressing specific surface or intracellular markers. The antibodies used included CD45 (APC-Cy7, 553082, BD Biosciences), CD3e (PE-Cy5.5, 551163, BD Biosciences), CD8α (APC, 100712, BioLegend), CD11c (FITC, 117306, BioLegend), XCR1 (APC, 148206, BioLegend), H-2K^b–SIINFEKL (PE, 17574382, Invitrogen), DNGR-1 (PE, 143503, BioLegend), H-2K^b (PE, 12-5958-82, eBioscience/Invitrogen), MHC-II (BV421, 562564, BD Biosciences), CD80 (APC, 560016, BD Biosciences), and CD86 (APC, 105012, BioLegend). Relevant isotype controls and, where appropriate, fluorescence-minus-one controls were used to define gates and assess background staining. For quantitative presentation, MFI values were normalized to the corresponding control group where indicated, including H-2K^b–SIINFEKL, H-2K^b, MHC-II, CD80 and CD86.

Immunofluorescence Staining

Cellular Immunofluorescence: MutuDCs were seeded on glass slides and treated with 5 μM fangchinoline for 24 hours, with or without the Syk inhibitor R406. Cells were fixed with 4% paraformaldehyde for 20 minutes at room temperature, washed with cold PBS, and blocked with 5% bovine serum albumin (BSA) for 2 hours. Cells were then incubated overnight at 4 °C with the primary antibody against Galectin-3 (89572S, CST) in a humidified chamber. After washing, cells were incubated with goat anti-rabbit Alexa Fluor 594-conjugated secondary antibody (ab150080, Abcam) for 1 hour at room temperature. Nuclei were counterstained with Hoechst 33342, and slides were sealed with coverslips for imaging using a laser confocal microscope.

Tissue Immunofluorescence: Tumor tissues were fixed in 4% paraformaldehyde at 4 °C for 24 hours, followed by dehydration, paraffin embedding, and sectioning at 4 μm. Sections were dewaxed at 65 °C, treated with 3% hydrogen peroxide to block endogenous peroxidase activity, and blocked with 5% BSA. Primary antibodies against CD8α (14-0081-82, Thermo Fisher), granzyme B (YT6137, Immunoway), and IFN-γ (YT2279, Immunoway) were applied overnight at 4 °C. After washing, sections were incubated with goat anti-rat Alexa Fluor 594 (ab150160, Abcam) and goat anti-rabbit Alexa Fluor 488 (4412S, CST) secondary antibodies (1:200 dilution) for 1 hour. Nuclei

were counterstained with Hoechst 33342, and slides were mounted with antifade reagent before imaging with a Zeiss fluorescence microscope.

Immunohistochemistry

Paraffin-embedded tumor sections were dewaxed at 65 °C and subjected to antigen retrieval in 10 mM trisodium citrate buffer (pH 6.0) at 100 °C for 10 minutes. After rinsing with reverse osmosis (RO) water, sections were treated with 3% hydrogen peroxide for 10 minutes to quench endogenous peroxidases and washed three times with PBS. Blocking was performed with 5% BSA for 15 minutes. Sections were incubated overnight at 4 °C with CD8α monoclonal antibody (53-6.7, eBioscience™, Thermo Fisher, 14-0081-82) at a 1:200 dilution. After washing, an HRP-conjugated secondary antibody was applied for 1 hour at 37 °C, and the signal was developed using DAB chromogen (ZLI-9018, ZSGB-BIO). Slides were counterstained with hematoxylin, dehydrated through graded ethanol, and imaged under a bright-field microscope.

Western Blot Analysis

Protein samples were resolved by SDS-PAGE and transferred to PVDF membranes using a constant current of 300 mA. Membranes were blocked with 5% skim milk in TBST for 2–4 hours at room temperature to minimize non-specific binding. Primary antibodies were incubated overnight at 4 °C, including IRF8 (18977-1-AP, proteintech), Galectin-3 (89572S, CST), phospho-Syk (Tyr352, YP0500; Tyr348, YP0614; Immunoway), total Syk (YT6110, Immunoway), β-actin (3700S, CST), and DNGR-1 (YN4563, Immunoway) at 1:1500 dilution. After washing, membranes were incubated with HRP-conjugated secondary antibodies for 1 hour at room temperature. Protein bands were visualized using an enhanced chemiluminescence (ECL) system and imaged with an automated imaging platform.

Molecular Docking Analysis

Molecular docking was used to evaluate the interaction between fangchinoline and DNGR-1. The crystal structure of DNGR-1 was obtained from the Protein Data Bank (PDB ID: 3J82), and the molecular structure of fangchinoline was retrieved from PubChem. Ligand and protein preparation were performed using PyMOL (v4.3.0) and AutoDockTools, including hydrogenation, charge assignment, and atom type specification (AD4 format). Docking simulations were conducted using AutoDock Vina (v1.1.2), with the receptor treated as rigid and the ligand flexible. The docking grid box was defined to encompass the potential binding site. Binding affinities and configurations were analyzed, and interaction forces were visualized using PyMOL and Discovery Studio, providing both 2D and 3D structural insights.

Plasmid Construction and Transfection

Plasmid vectors used in this study were obtained from VectorBuilder (details in Table S5). Plasmids were amplified in LB broth containing penicillin and incubated at 37 °C overnight with shaking. DNA was extracted and adjusted to a final concentration of 200 ng/μL. For lentiviral packaging, 293T cells were co-transfected with the target plasmid and helper plasmids psPAX2 and pMD2.G using calcium phosphate precipitation. After 6 hours of transfection, the medium was replaced with complete DMEM, and viral supernatants were collected at 48 hours and concentrated. MutuDCs were transduced with viral particles over two consecutive 24-hour infections. After infection, transduced cells were selected with puromycin to establish stable cell lines for downstream functional assays.

Synthesis of the Fan-BP Molecular Probe (Fan-probe)

The Fan-BP molecular probe was synthesized to facilitate targeted binding studies. The probe was constructed by conjugating a bromide-terminated short polyethylene glycol (PEG) chain to a benzophenone (BP) analog containing a propyne group. This was achieved via a Williamson etherification reaction, enabling the coupling of the methylene bromide group on the PEG chain with the phenolic hydroxyl group of fangchinoline. The resulting product, termed Fan-BP (Fan-probe), was designed for use in photoaffinity labeling and binding assays. A structurally similar control probe (Ctrl-probe) was synthesized for comparative analysis. The chemical structures of both probes are shown in Fig. S6.

Supplementary Figures

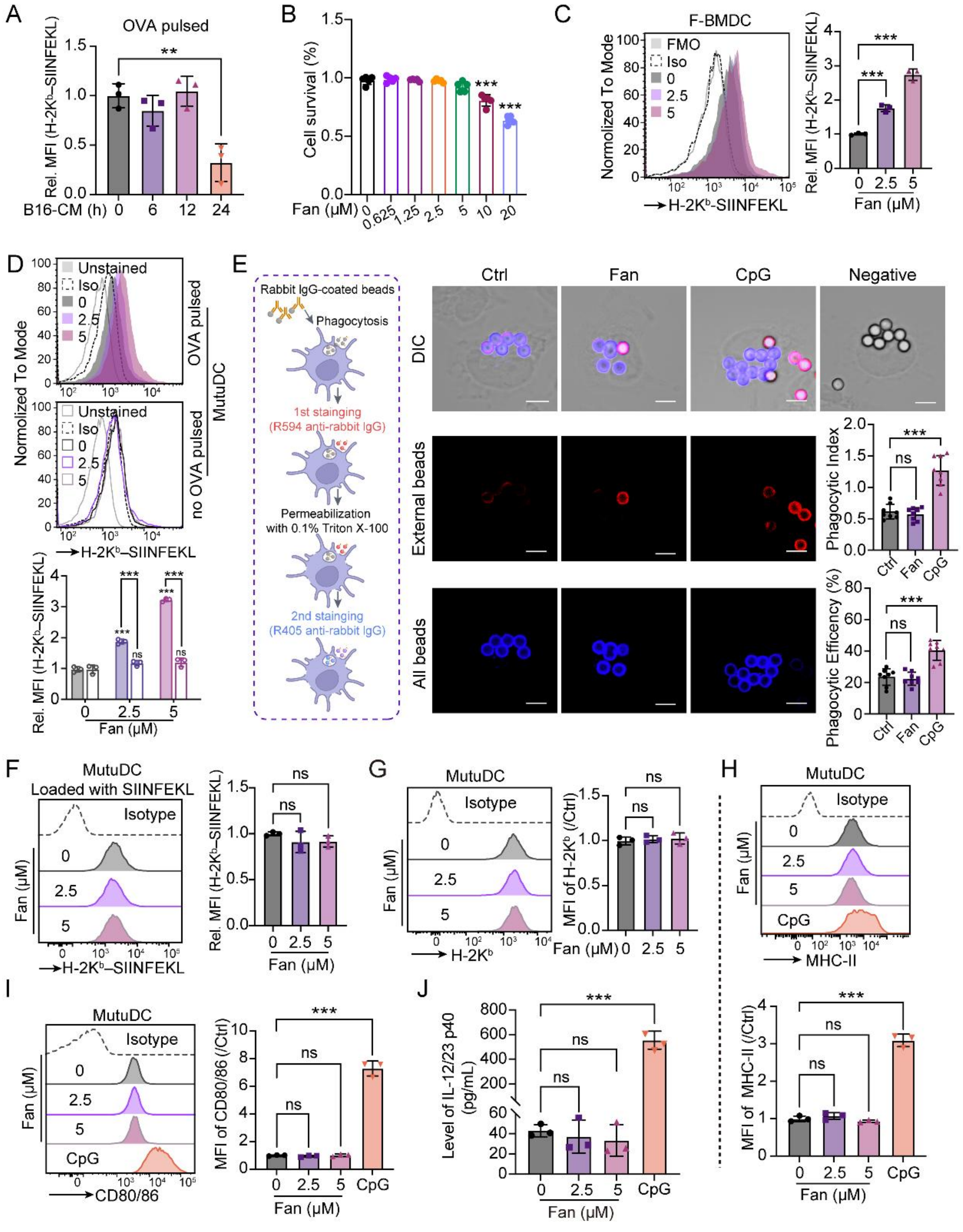

Fig. S1. Fangchinoline selectively enhances dendritic cell antigen cross-presentation without inducing co-stimulatory or inflammatory activation. (A) Exposure of MutuDCs to B16 tumor-conditioned medium impaired antigen cross-presentation. Cells were cultured for 24 h in a 1:1 mixture of complete medium and B16 cell supernatant in the presence of OVA protein (200 µg/mL), followed by staining with anti-mouse H-2K^b–SIINFEKL antibody and flow cytometric analysis of H-2K^b–SIINFEKL$^+$ cells. One-way ANOVA with Tukey's test (n=3). **(B)** Cytotoxicity of

fangchinoline in MutuDCs was assessed using a CCK-8 assay. Cells were treated with increasing concentrations of fangchinoline (0, 0.625, 1.25, 2.5, 5, 10, 20 μM) for 24 h, and cell viability was determined. One-way ANOVA with Tukey's test (n=3). **(C)** Flt3L-induced bone marrow-derived dendritic cells (F-BMDCs) were treated with fangchinoline (2.5 or 5 μM) and OVA protein (200 μg/mL) for 24 h. Antigen cross-presentation was assessed by flow cytometry using H-2K^b–SIINFEKL staining. One-way ANOVA with Tukey's test (n=3). **(D)** MutuDCs were treated with fangchinoline (2.5 or 5 μM) for 24 h without OVA stimulation. Cells were stained with an anti-mouse H-2K^b–SIINFEKL antibody, and the relative H-2K^b–SIINFEKL MFI was determined by flow cytometry. One-way ANOVA with Tukey's post hoc test (n=3). **(E)** MutuDCs treated with fangchinoline (5 μM) or CpG (1 μM) were incubated with IgG-opsonized polystyrene beads for 2 h, and phagocytosis was analyzed by confocal microscopy using an inside-out staining strategy to distinguish extracellular and internalized beads. Internalized beads were defined as Alexa Fluor 405 single-positive signals, whereas extracellular beads were identified as Alexa Fluor 594/Alexa Fluor 405 double-positive signals. For the negative control, beads were not incubated with rabbit IgG but were stained with the fluorescent secondary antibody in parallel. Phagocytosis was quantified as the phagocytic index, defined as the number of internalized beads per total number of cells in each field, and the phagocytic efficiency, defined as the percentage of cells containing internalized beads among total cells in each field. One-way ANOVA with Tukey's post hoc test (n = 8 randomly selected microscopic fields). Scale bar, 5 μm. **(F)** MutuDCs were treated with fangchinoline (2.5 or 5 μM) and OVA peptide (SIINFEKL, 2 μg/mL) for 24 h. Peptide presentation was assessed by flow cytometry using H-2K^b–SIINFEKL staining. One-way ANOVA with Tukey's test (n=3). **(G)** Surface expression of MHC class I (H-2K^b) on MutuDCs treated with fangchinoline (2.5 or 5 μM) for 24 h was analyzed by flow cytometry. One-way ANOVA with Tukey's test (n=3). **(H)** Expression of MHC class II (I-A/I-E) on MutuDCs treated with fangchinoline (2.5 or 5 μM) or the positive control CpG (0.5 μM) for 24 h was measured by flow cytometry. One-way ANOVA with Tukey's test (n=3). **(I)** Expression of CD80 and CD86 on MutuDCs treated as in (H) was analyzed by flow cytometry. One-way ANOVA with Tukey's test (n=3). **(J)** Secretion of the p40 subunit shared by IL-12 and IL-23 was measured by ELISA after treating MutuDCs with fangchinoline (2.5 or 5 μM) or CpG (0.5 μM) for 24 h. One-way ANOVA with Tukey's test (n=3). MFI, mean fluorescence intensity. * $P < 0.05$; ** $P < 0.01$; ***$P < 0.001$; ns: not significant.

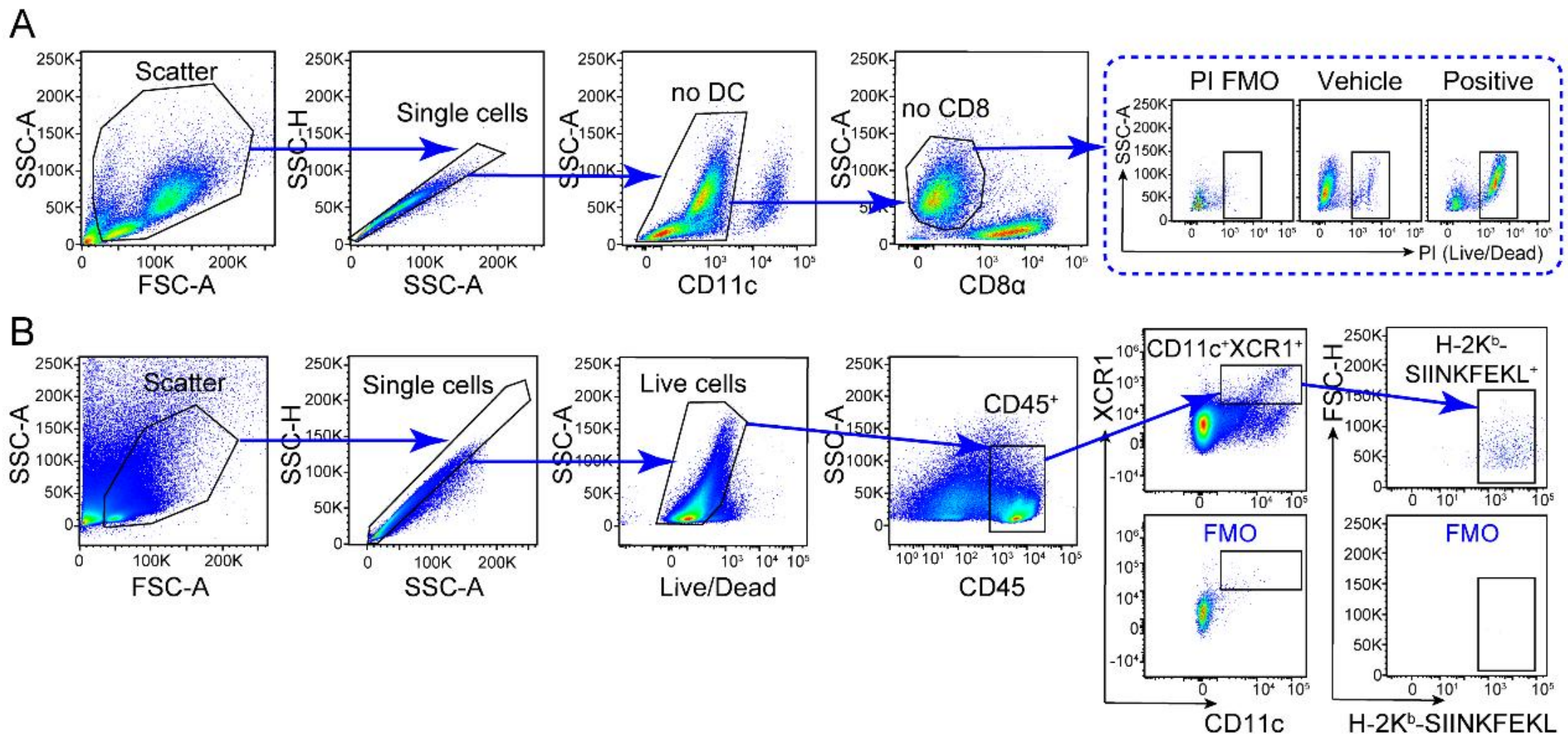

Fig. S2. Gating strategies for flow cytometric analysis. (A) Flow cytometry gating strategy used for the *in vitro* cytotoxicity assay shown in Fig. 1H. **(B)** Gating strategy for analysis of conventional type 1 dendritic cells (cDC1s) and SIINFEKL-presenting cDC1s (H-2K^{b}–SIINFEKL^{+}) in Fig. 1J–K. Fan, fangchinoline.

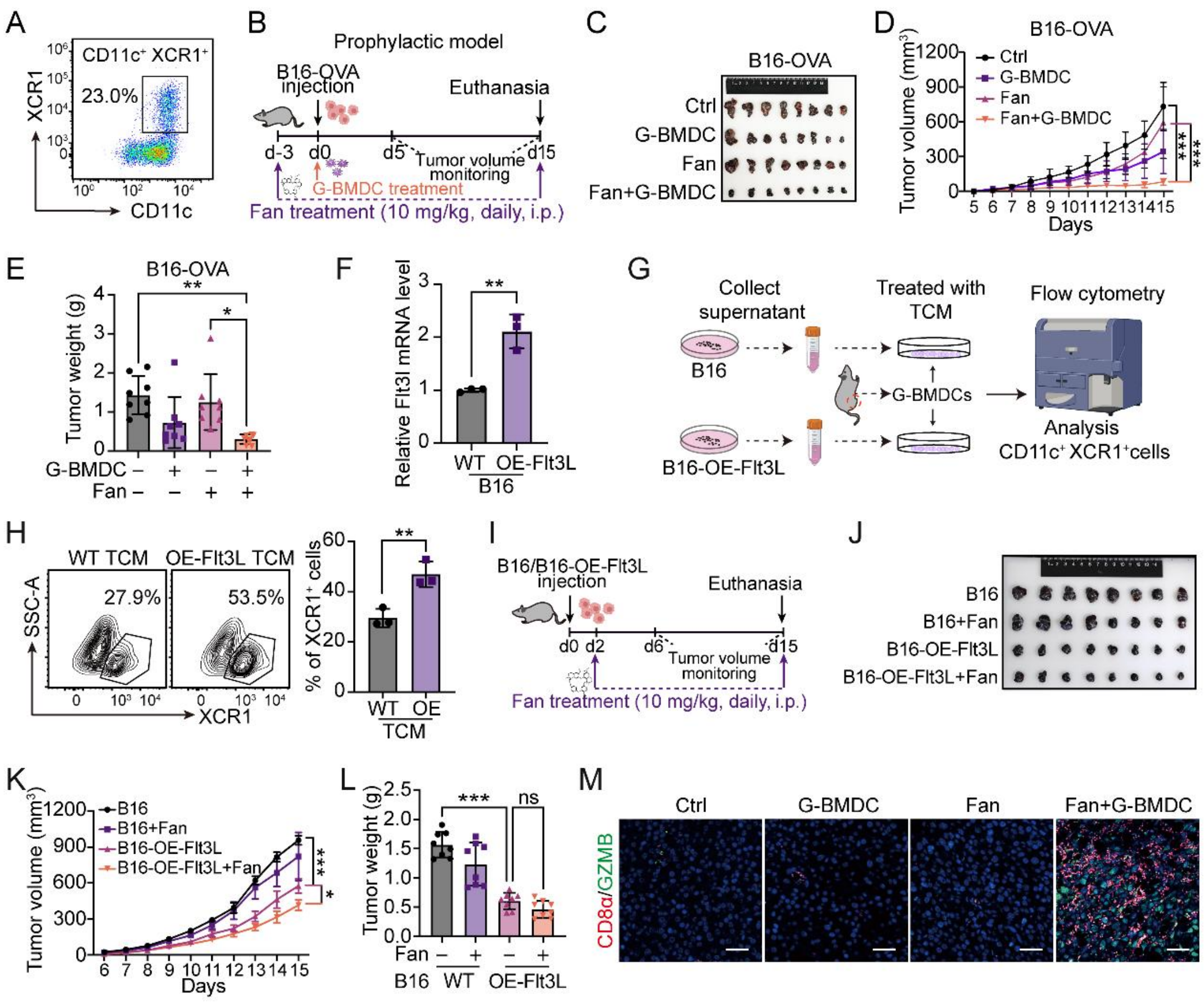

Fig. S3. Evaluation of prophylactic and therapeutic tumor responses to fangchinoline combined with G-BMDCs and Flt3L-overexpressing melanoma

cells. (A) Flow cytometric analysis of the frequency of $XCR1^+$ cells in the F-BMDC preparations used for adoptive transfer. **(B)** Schematic of the prophylactic treatment model. C57BL/6 mice were subcutaneously inoculated with 2×10^5 B16-OVA cells and 5×10^4 granulocyte-derived BMDCs (G-BMDCs). Fangchinoline (10 mg/kg/day) was administered intraperitoneally from day –3 to day 15. Experimental groups included: Control, G-BMDC, fangchinoline, and fangchinoline + G-BMDC. Tumors were collected on day 15. **(C-E)** Representative tumor images, tumor growth curves, and tumor weights from the prophylactic model. Two-way ANOVA with Tukey's post hoc test (n=8). **(F)** Flt3L expression in the B16-OE-Flt3L cell line was confirmed by qPCR. Analyzed by two-tailed unpaired t-test (n=3). **(G)** Schematic of the experimental procedure. The function of an Flt3L-overexpressing B16 melanoma cell line (B16-OE-Flt3L) was assessed by treating BMDCs with tumor-conditioned medium (TCM), followed by flow cytometric analysis of $CD11c^+$ $XCR1^+$ cells. **(H)** BMDCs were cultured with tumor-conditioned medium (TCM) prepared from B16 or Flt3L-overexpressing B16 cells (B16-OE-Flt3L), mixed 1:1 with BMDC complete medium. The proportion of $XCR1^+$ BMDCs was analyzed by flow cytometry. Analyzed by two-tailed unpaired t-test (n=3). **(I)** Schematic of the therapeutic treatment model. C57BL/6 mice were subcutaneously inoculated with 2×10^5 B16 or B16 OE-Flt3L cells, followed by daily intraperitoneal injections of fangchinoline (10 mg/kg/day) from day 2 to day 15. Experimental groups included: B16, B16 + fangchinoline, B16-OE-Flt3L, and B16-OE-Flt3L + fangchinoline. Tumors were collected on day 15. **(J-L)** Representative tumor images, tumor growth curves, and tumor weights from the therapeutic model. Two-way ANOVA with Tukey's post hoc test (n=8). **(M)** Immunofluorescence analysis of B16 tumor tissues stained for CD8α (red) and granzyme B (GZMB, green). Nuclei were counterstained with Hoechst 33342. Scale bars, 50 μm. Groups: Control, G-BMDC, fangchinoline, and fangchinoline + G-BMDC. Fan, fangchinoline. TCM, tumor-conditioned medium. $^*P < 0.05$; $^{**} P < 0.01$; $^{***}P < 0.001$; ns: not significant.

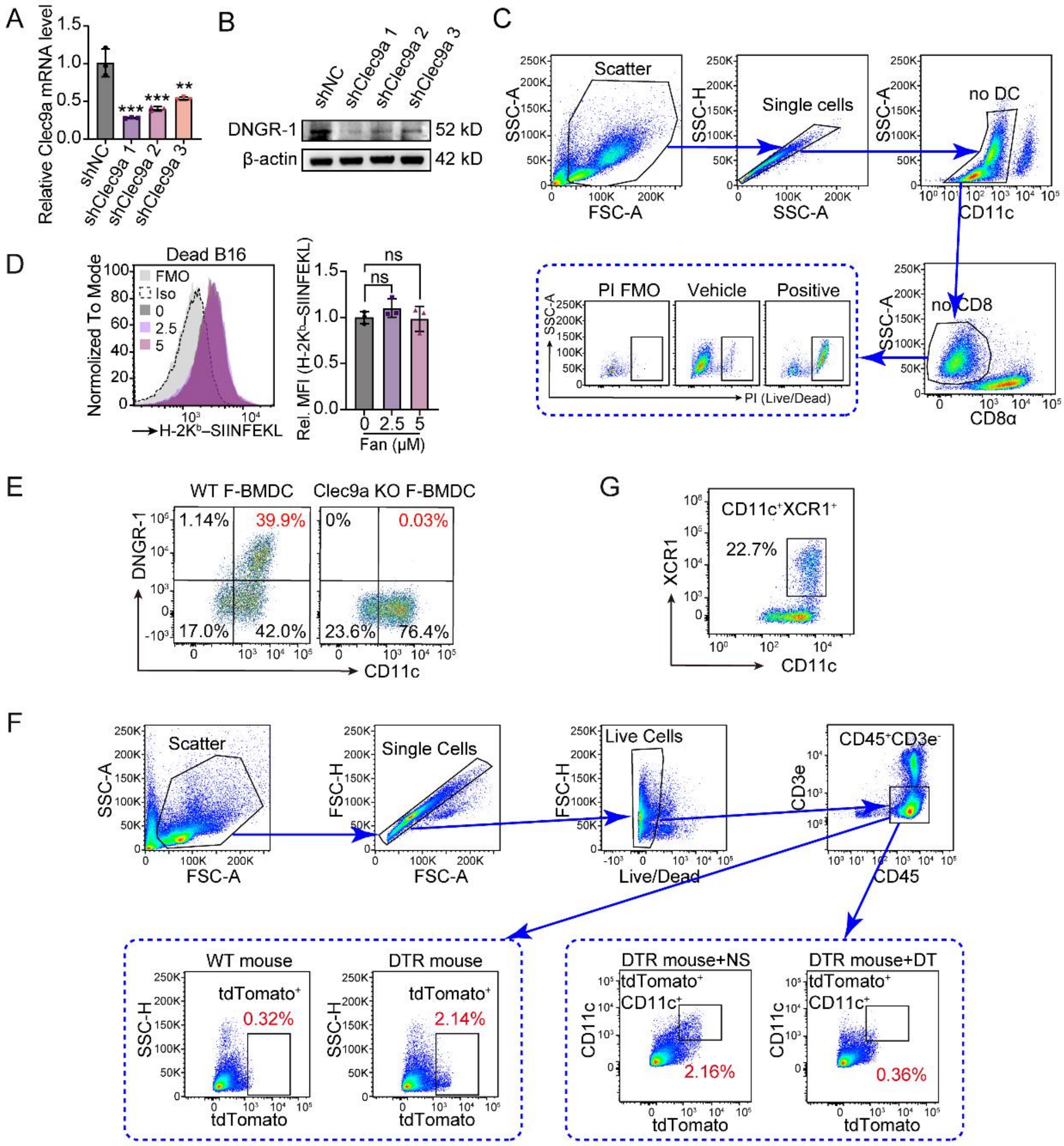

Fig. S4. Validation of DNGR-1/Clec9a-deficient dendritic cell models and flow cytometric analysis of antigen presentation and cytotoxicity. (A-B) Knockdown efficiency of three independent shClec9a MutuDC cell lines was validated by qPCR and western blot. One-way ANOVA with Tukey's test (n=3). **(C)** Flow cytometry gating strategy for evaluating cytotoxicity in the fangchinoline and αDNGR-1 combination treatment. After doublet exclusion, $CD11c^{+}$ dendritic cells and $CD8^{+}$ T cells were gated out. The remaining $CD11c^{-}CD8^{-}$ population—enriched for target tumor cells—was analyzed for apoptosis using PI staining. **(D)** Dead B16 cells were used as an antigen specificity control lacking OVA. MutuDCs were co-incubated with these dead B16 cells and treated with fangchinoline (2.5 or 5 μM) for 24 h. Surface $H\text{-}2K^{b}$–SIINFEKL MFI was measured by flow cytometry and normalized to the control, providing a negative control for OVA-specific 25-D1.16 staining in the dead-cell antigen setting. One-way

ANOVA with Tukey's post hoc test (n=3). **(E)** DNGR-1 expression in Flt3L-induced BMDCs from wild-type and Clec9a knockout (KO) mice was analyzed by flow cytometry. **(F)** Flow cytometric analysis of tdTomato expression in splenocytes from wild-type (WT; C57BL/6J) and CD11c-tdTomato-DTR (DTR) mouse. The frequency of CD11c⁺ tdTomato⁺ cells was further assessed in DTR mouse treated with either normal saline (NS) or diphtheria toxin (DT; 30 ng/g). Representative gating strategy and quantification of tdTomato⁺ and CD11c⁺ tdTomato⁺ populations are shown. **(G)** Flow cytometric analysis of the frequency of XCR1⁺ cells in the F-BMDC preparations used for adoptive transfer. Fan, fangchinoline; F-BMDC, Flt3L-induced bone marrow–derived dendritic cells; MFI, mean fluorescence intensity. ** P < 0.01; ***P < 0.001; ns: not significant.

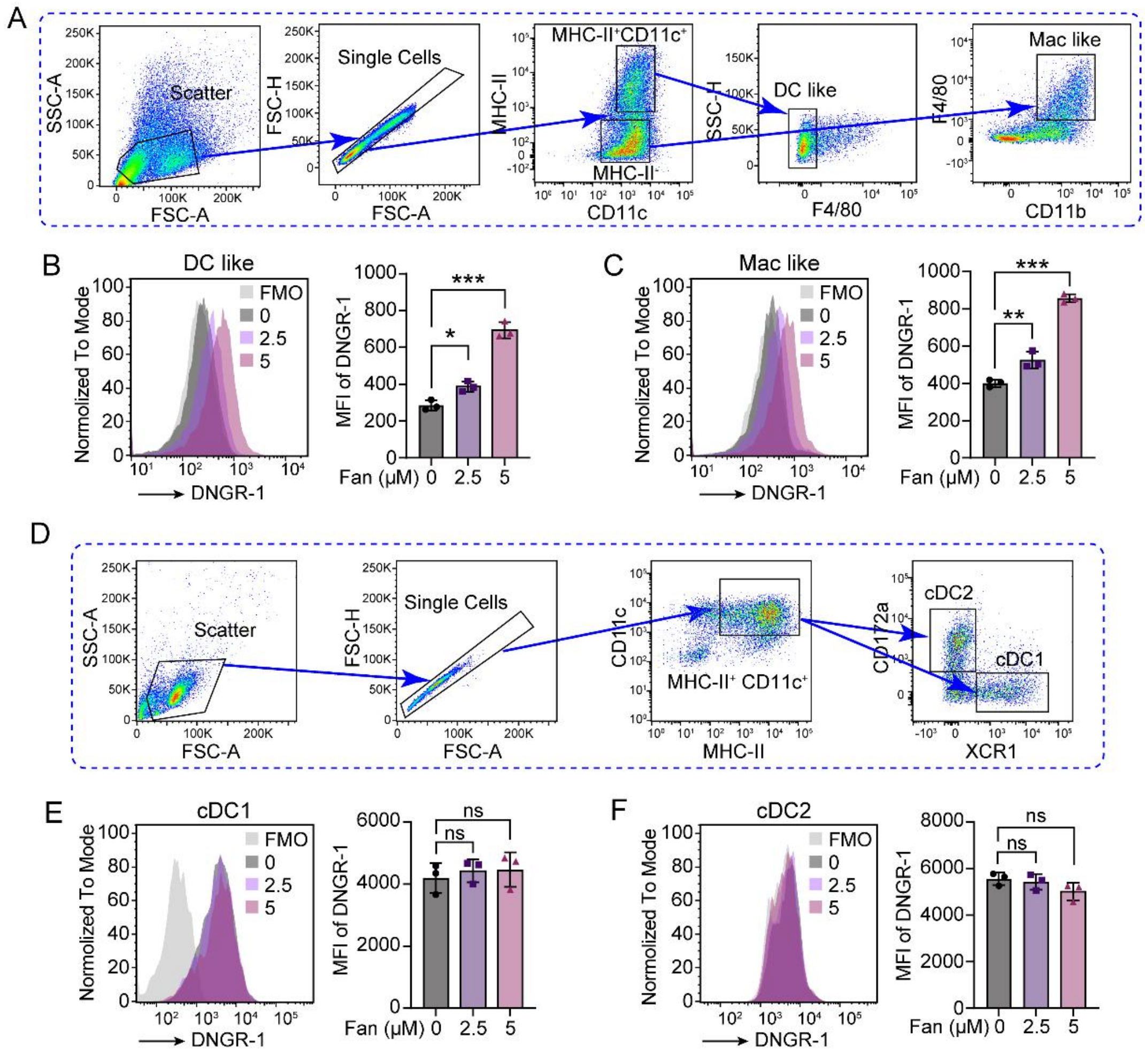

Fig. S5. Differential effect of fangchinoline on DNGR-1 expression in GM-CSF/IL-4–derived versus Flt3L-derived DC cultures. **(A)** Gating strategy for analysis of DC-like and macrophage (Mac)-like populations in GM-CSF/IL-4–derived cultures. **(B–C)** Using the gating strategy shown in (A), DNGR-1 MFI was assessed in the DC-like and Mac-like compartments by flow cytometry. **(D)** Gating strategy for cDC1 and

cDC2 subsets in Flt3L-derived DC cultures. **(E–F)** Using the strategy shown in (D), DNGR-1 MFI was measured by flow cytometry in cDC1 and cDC2 subsets. Fan, fangchinoline; Mac, macrophage; MFI, mean fluorescence intensity. * P < 0.05; ** P < 0.01; ***P < 0.001; ns: not significant.

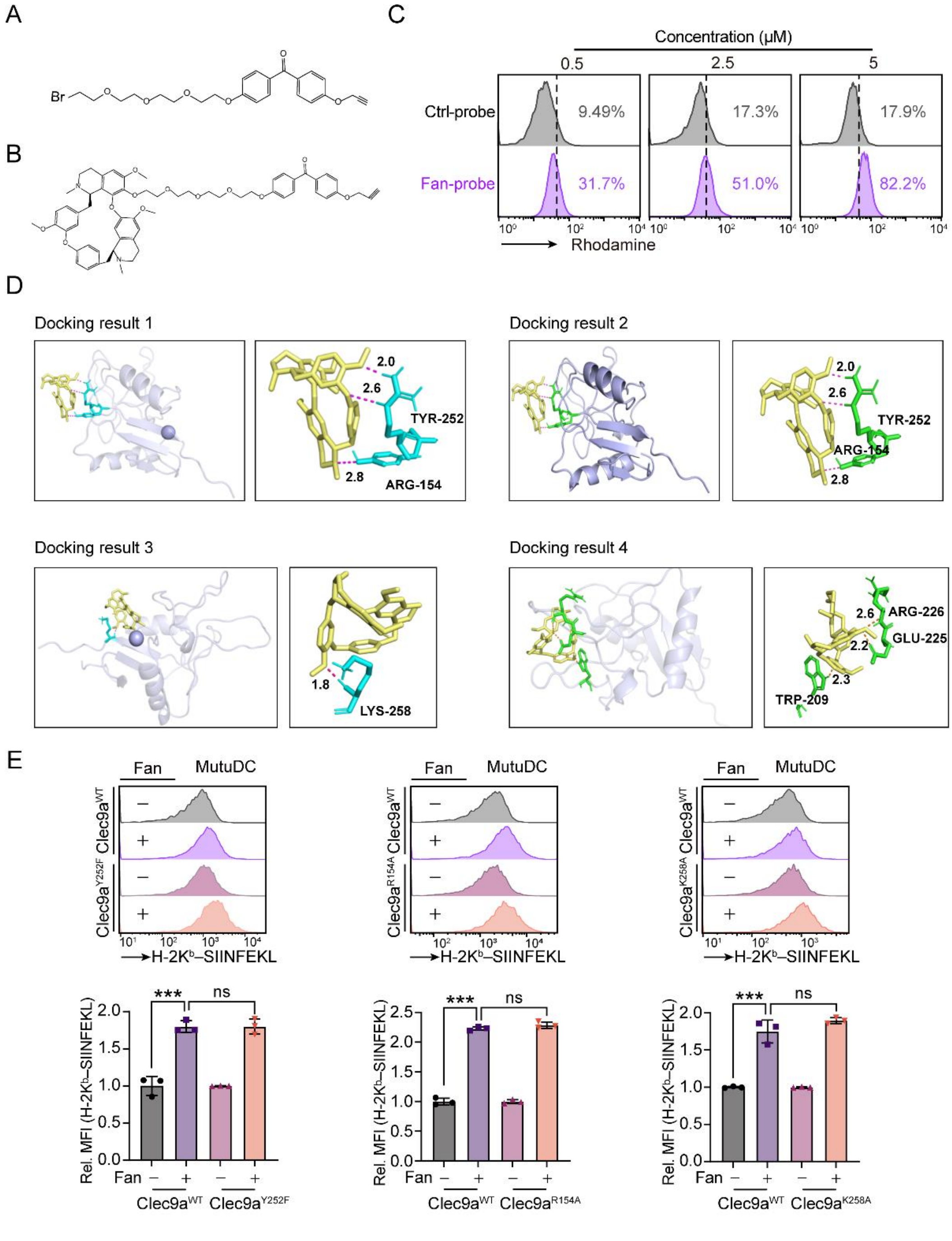

Fig. S6. Characterization of fangchinoline binding to DNGR-1 using a custom chemical probe and site-directed mutagenesis. (A-B) Chemical structures of the control probe (Ctrl-probe, A) and the fangchinoline-conjugated probe (Fan-probe, B).

(C) Flow cytometric analysis of Fan-probe and Ctrl-probe binding to MutuDCs at increasing concentrations (0.5, 2.5, and 5 μM). Cells were incubated with probes and labeled via click chemistry using a rhodamine fluorophore. Binding efficiency was quantified by the percentage of rhodamine-positive cells, indicating probe-target interactions. **(D)** Molecular docking analysis of fangchinoline binding to DNGR-1 (PDB ID: 3J82). Docking result 1: Binding at Arg154 and Tyr252 (binding energy: –6.75 kcal/mol). Docking result 2: Alternative pose at the same site involving Arg154 and Tyr252 (binding energy: –6.74 kcal/mol). Docking result 3: Binding near Lys258 (binding energy: –7.59 kcal/mol). Docking result 4: Binding at a composite pocket formed by Trp209, Glu225, and Arg226 (binding energy: –9.25 kcal/mol). All predicted interactions involved hydrogen bonding only. **(E)** Functional validation of predicted binding residues via site-directed mutagenesis. Mutations were introduced in DNGR-1 at Tyr252, Arg154, and Lys258 in MutuDCs. Cells expressing wild-type or mutant DNGR-1 were treated with or without fangchinoline and incubated with OVA (200 μg/mL). Antigen cross-presentation was assessed by flow cytometry using H-2K^b–SIINFEKL staining. Surface H-2K^b–SIINFEKL MFI was normalized to the control. Two-way ANOVA with Tukey's post hoc test (n=3). Fan, fangchinoline; Ctrl-probe, control probe; WT, wild-type; *P < 0.05; ** P < 0.01; ***P < 0.001; ns: not significant.

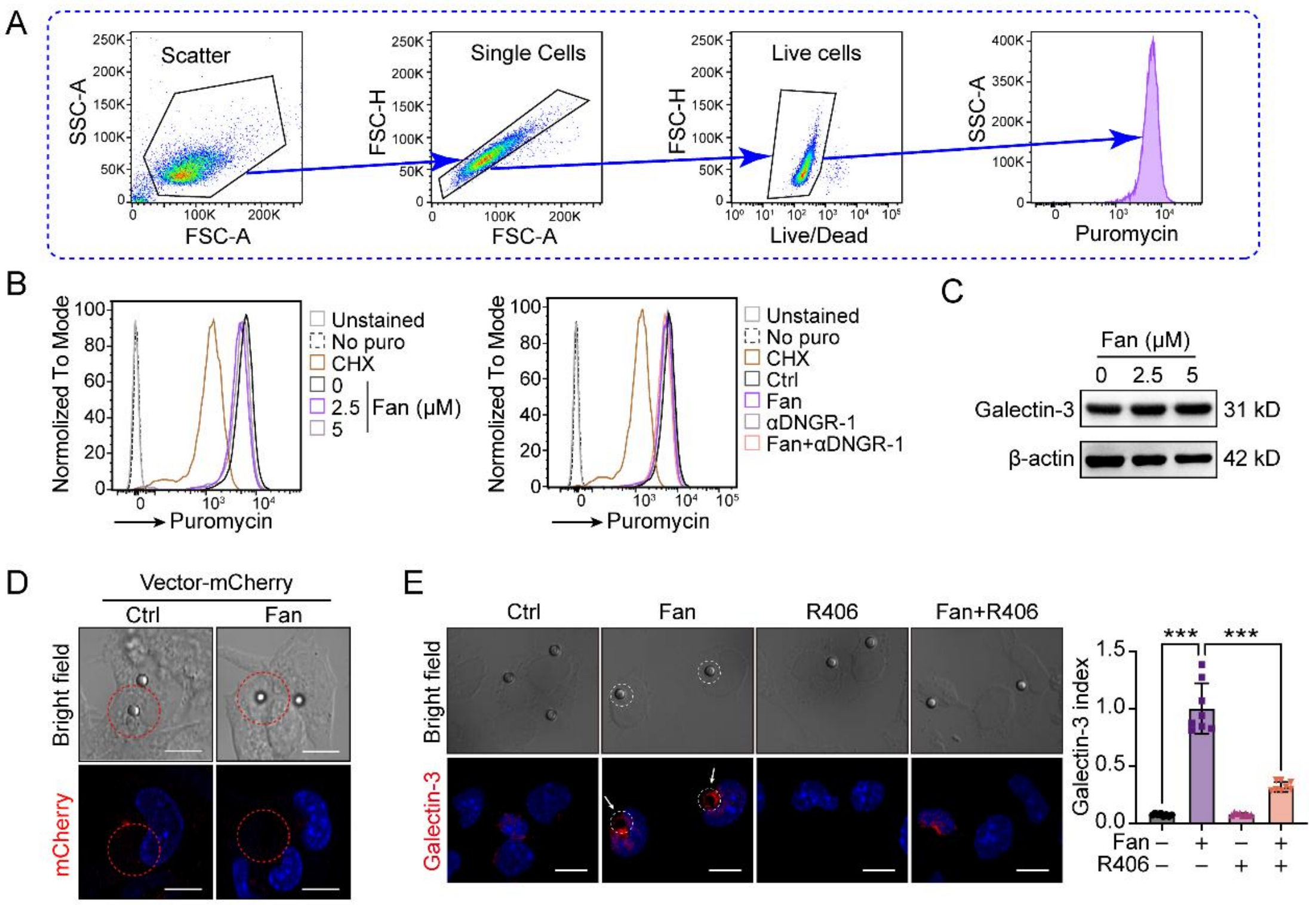

Fig. S7. Flow cytometric gating strategy, specificity controls for puromycin labeling, and validation of mCherry–lysenin fluorescence. (A) Gating strategy used for flow cytometric analysis of puromycin-labeled cells. **(B)** MutuDCs were stained with an anti-puromycin antibody and analyzed by flow cytometry under non-

permeabilized conditions without saponin. Groups included control, fangchinoline treatment, and DNGR-1 blockade (αDNGR-1). Additional control groups included unstained cells, cells not treated with puromycin (no puro), and cells treated with cycloheximide (CHX). **(C)** Western blot analysis of Galectin-3 expression in MutuDCs treated with fangchinoline (2.5 or 5 μM). **(D)** A stable dendritic cell line expressing vector-encoded mCherry alone was used as a negative control to evaluate the specificity of the mCherry–lysenin signal. Cells were treated with or without fangchinoline (5 μM) for 24 h. No appreciable red fluorescence was detected around phagosomes under either condition, confirming that fangchinoline does not induce non-specific fluorescence and that mCherry alone does not localize to phagosomal membranes. Scale bars, 5 μm. Nuclei were counterstained with Hoechst 33342. **(E)** Immunofluorescence analysis of Galectin-3 (red) recruitment to phagosomes in MutuDCs treated with fangchinoline (5 μM), Syk inhibitor R406 (1 μM), or both. Cells were pulsed with 3.00 μm amino microspheres to label phagosomes, followed by 24 h incubation with indicated treatments. Galectin-3 accumulation surrounding phagosomes was quantified using ImageJ as a readout of phagosomal membrane damage. Scale bars, 5 μm. Nuclei were counterstained with Hoechst 33342. Data analyzed by two-way ANOVA with Tukey's post hoc test (n=3). Fan, fanchinoline; ***P < 0.001.

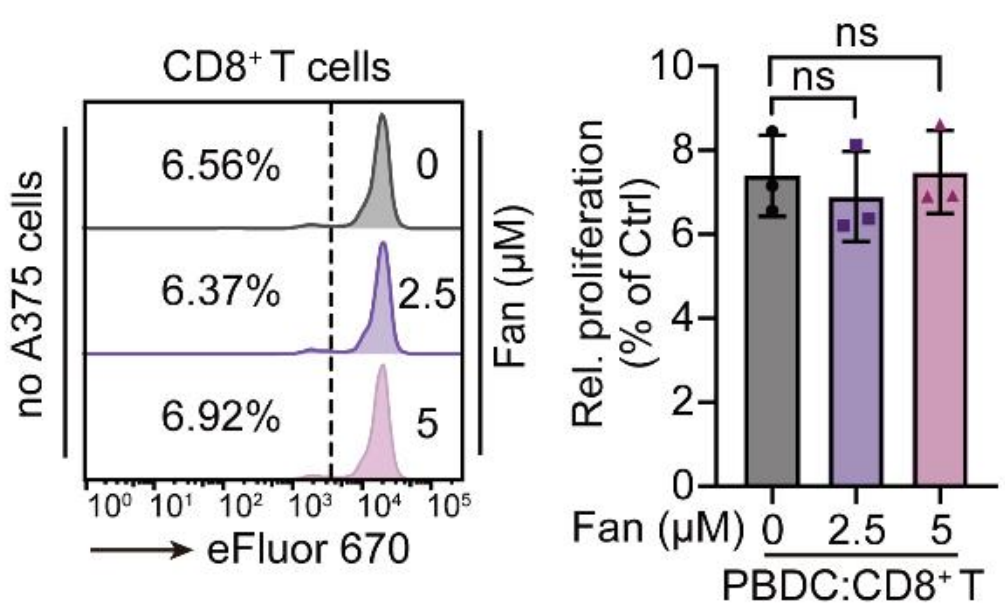

Fig. S8. Fangchinoline does not induce non-specific T cell proliferation. To confirm that T cell proliferation induced by monocyte-derived dendritic cells (moDCs) was antigen-specific, moDCs without A375 tumor cell lysate loading were co-cultured with autologous $CD8^+$ T cells under the same conditions. T cell proliferation was assessed using the eFluor™ 670 dilution method. Minimal $CD8^+$ T cell proliferation was observed before and after fangchinoline treatment.

Supplementary Tables

Table S1. PCR primer sequences used for genotyping transgenic mice.

Primer name	Primer sequence	Primer Type
mClec9a-P1	CATTTCAAGGTTTCAGGGATGACC	Homozygote
mClec9a-P2	TGTTTCAACAATTCCCAGGAAGGT	Homozygote
mClec9a-P3	TGTCACATAAAACACCATTTCAAGG	Wild type Forward
mClec9a-P4	GCATGGGCTATATCTGACTTGGA	Wild type Reverse
mDTR-P1	GCAGGGCCTCTCACTTGAAT	Wild type Forward
mDTR-P2	GGGCCACAAGCTGACTATGT	Wild type Reverse
mDTR-P3	AGCTAGCCACCTTGCTTCAG	Mutant Forward
mDTR-P4	TTGTAATCGGGGATGTCGGC	Mutant Reverse
mDTR-P5	CAAATGTTGCTTGTCTGGTG	Internal Positive
mDTR-P6	GTCAGTCGAGTGCACAGTTT	Internal Positive

Table S2. PCR array analysis of gene expression in fangchinoline-treated MutuDCs.

Target Name	Fan/Control	Target Name	Fan/Control
Ccl11	Undetermined	Il12b	0.842497713
Ccl19	Undetermined	Tlr1	0.844321933
Cd209a	Undetermined	Itgb2	0.847407306
Cd28	Undetermined	Nfkb1	0.868740421
Cd40lg	Undetermined	Lyn	0.875404403
Csf3	Undetermined	Ccl3	0.881921267
Cxcl1	Undetermined	Ccl12	0.88873923
Fas	Undetermined	Tlr9	0.891872533
Ifng	Undetermined	Erbb2	0.915886941
Il12a	Undetermined	Cd80	0.930377024
Il2	Undetermined	Il16	0.948501075
Ccl17	0.363009753	Cebpa	0.952657092
CD8B	0.393234925	Rela	0.95394263
Cd8a	0.474707836	Ccr1	0.95721216
Ccr5	0.481180053	HPRT1	0.979633507
Relb	0.49618065	Cd36	0.987986353

Table S2. PCR array analysis of gene expression in fangchinoline-treated MutuDCs. (continued)

Target Name	Fan/Control	Target Name	Fan/Control
Cxcl10	0.533149231	Itgam	1.001134983
Ccl4	0.544764618	Rac1	1.00249124
Ccl5	0.55460272	Icam2	1.032151179
Flt3	0.590310465	Csf1r	1.058832039
Cxcl12	0.607963581	Ccr3	1.073272523
Fcer2a	0.621147307	Fcer1a	1.082940512
Ccr9	0.665574489	Cxcr1	1.088599111
Cd86	0.686993475	Stat3	1.106143325
Fcgrt	0.687392315	Il10	1.109674803
CIITA	0.692170871	Ccr2	1.117523387
Il6	0.705137003	CTSL1	1.168945998
Ptprc	0.71933725	Csf2	1.181848409
Cxcl2	0.725188751	Flt3l	1.203381146
CREB1	0.731460069	Rag1	1.215496416
Cd74	0.733340966	Cxcr4	1.22178142
Ccl7	0.734472795	Cdkn1a	1.253717176
Tgfb1	0.758702591	Lrp1	1.269522501
Tap2	0.760534381	Irf7	1.326847765
Cd40	0.762806135	Tlr7	1.400861934
H2-Dma	0.765207076	GAPDH	1.500141165
Tapbp	0.76735176	Thbs1	1.671465599
Clec4b2	0.775054677	Cd1d1	1.902034797
Ccl2	0.782982932	Csf1	1.946052064
Cd44	0.793529154	Cd2	1.968062617
CTSB	0.796039425	Fcgr1	2.010094299
Cd4	0.807653677	Mif	2.320484467
Icam1	0.818532195	Tlr2	3.340664712
Tnf	0.819267312	Ccl8	3.905887939
Cdc42	0.839031896	Clec9a	6.799836228
Ccl20	0.839817621	Irf8	4.419857939

Table S3. shRNA target sequences for mouse DNGR-1 (Clec9a).

Name	Target sequence
mClec9a[shRNA_1]	CCTCACAGATATAGAGATTTA
mClec9a[shRNA_2]	GCTTGTTAGCAACGTCCATTT
mClec9a[shRNA_3]	TGAACTAAACAGCCCATTTAA

Table S4. Primer sequences for qPCR.

Serial number	Primer name	Primer sequence
1	mClec9a-F	TCTTGTCTTGGAGCAGCAGG
2	mClec9a-R	TCTCTGCAGTAAGGCTTGGC
3	mIrf8-F	CGGGGCTGATCTGGGAAAAT
4	mIrf8-R	CACAGCGTAACCTCGTCTTC
5	mFlt3l-F	TGGCCGTCAATCTTCAGGAC
6	mFlt3l-R	TCCTCCAGAAGCGTTTGCAT

Table S5. Plasmid constructs used in this study.

Serial number	Plasmid vector name
1	pLV[Exp]-Puro-EF1A>mFlt3l[NM_001402831.1]
2	pLV[shRNA]-Neo-U6>Scramble[shRNA#1]-U6>Scramble[shRNA#2]
3	pLV[shRNA]-Neo-U6>{mClec9a[shRNA_1]}
4	pLV[shRNA]-Neo-U6>{mClec9a[shRNA_2]}
5	pLV[shRNA]-Neo-U6>{mClec9a[shRNA_3]}
6	pLV[Exp]-Puro-EF1A>mCherry
7	pLV[Exp]-Puro- EF1A>{lysenin(Mouse_co)}:3xGGGGS:mCherry
8	pLV[Exp]-Puro-EF1A>mClec9a[NM_001205363.1]
9	pLV[Exp]-Puro-EF1A>{mClec9a[NM_001205363.1]*(Y252F)}
10	pLV[Exp]-Puro-EF1A>{mClec9a[NM_001205363.1]*(R154A)}
11	pLV[Exp]-Puro-EF1A>{mClec9a[NM_001205363.1]*(K258A)}
12	pLV[Exp]-Puro-EF1A>{mClec9a[NM_001205363.1]*(W209F)}
13	pLV[Exp]-Puro-EF1A>{mClec9a[NM_001205363.1]*(R226A)}
14	pLV[Exp]-Puro-EF1A>{mClec9a[NM_001205363.1]*(E225Q)}
